# Multiscale spatial relationship‐based model for predicting bladder wall dose in pelvic radiotherapy

**DOI:** 10.1002/acm2.14153

**Published:** 2023-09-12

**Authors:** Xiang Gao, Lei Ge, Junfeng Gao, Zheng Cao

**Affiliations:** ^1^ Oncology Department Hefei First People's Hospital Hefei China; ^2^ National Synchrotron Radiation Laboratory University of Science and Technology of China Hefei China

**Keywords:** bladder wall dose, multiple linear regression, multiscale spatial relationship, pelvic tumors, prediction model, radiotherapy

## Abstract

**Purpose:**

This research aimed to develop a prediction model to assess bladder wall dosimetry during radiotherapy for patients with pelvic tumors, thereby facilitating the refinement and evaluation of radiotherapy treatment plans to mitigate bladder toxicity.

**Methods:**

Radiotherapy treatment plans of 49 rectal cancer patients and 45 gynecologic cancer patients were collected, and multiple linear regression analyses were used to generate prediction models for bladder wall dose parameters ($V_{10-45Gy\;}\left(cm^{3}\right)$, $D_{mean}\left(\mathrm{Gy}\right)$). These models were based on the multiscale spatial relationship between the planning target volume (PTV) and the bladder or bladder wall. The proportion of bladder or bladder wall volume overlapped by the different distance expansions of the PTV was used as an indicator of the multiscale spatial relationship. The accuracy of these models was verified in a cohort of 12 new patients, with further refinement of radiotherapy treatment plans using the predicted values as optimization parameters. Model accuracy was assessed using root mean square error (RMSE) and mean percentage error (MPE).

**Results:**

Models derived from individual disease data outperformed those derived from combined datasets. Predicted bladder wall dose parameters were accurate, with the majority of initial calculated values for new patients falling within the 95% confidence interval of the model predictions. There was a robust correlation between the predicted and actual dose metrics, with a correlation coefficient of 0.943. Using the predicted values to optimize treatment plans significantly reduced bladder wall dose (*p* <0.001), with bladder wall $D_{\mathrm{mean}}\left(Gy\right)$ and $V_{10-45Gy\;}\left(cm^{3}\right)$ decreasing by 2.27±0.80 Gy (5.8%±1.8%) and 2.96±2.05 cm^3^ (7.9%±5.4%), respectively.

**Conclusion:**

The formulated prediction model provides a valuable tool for predicting and minimizing bladder wall dose and for optimizing and evaluating radiotherapy treatment plans for pelvic tumor patients. This approach holds promise for reducing bladder toxicity and potentially improving patient outcomes.

## 1 INTRODUCTION

External beam radiotherapy is an important treatment option for pelvic cancer. Modern radiotherapy techniques have allowed more precise dose distribution in pelvic tumor radiotherapy, reducing the exposure of normal tissues to x‐rays. However, healthy bladder tissue inevitably receives radiation, which can lead to acute urologic injury during early radiation therapy.^1^, ^2^, ^3^ Furthermore, exfoliation, ulceration, and necrosis of the urethral epithelium, along with dilation and fibrosis of the submucosal capillaries, can cause progressive and irreversible urologic damage approximately 6 months after radiotherapy.^1^, ^2^, ^3^ Studies have reported that a significant number of pelvic cancer patients develop acute bladder toxicity reactions during treatment, ranging from 29% to 50%.^4^, ^5^, ^6^, ^7^ In addition, the incidence of severe urinary radiation injury may be more than 20% in long‐term survivors of pelvic cancer.^6^, ^7^, ^8^ Therefore, it is essential to investigate the factors that cause urologic radiation injury and to reduce its incidence to improve the quality of life of pelvic cancer patients.

Previous studies have shown that urologic radiation injury is primarily related to the dose to the bladder wall.^8^, ^9^, ^10^, ^11^, ^12^, ^13^ Therefore, bladder wall contouring and bladder wall dose minimization during radiotherapy planning are necessary to reduce bladder toxicity in patients with pelvic tumors. However, in the clinic, designing a radiotherapy treatment plan for each patient that achieves an optimal bladder wall dose distribution is challenging due to the experience of the medical physicist, the complexity of the plan, and the time urgency of designing the plan. It is also difficult for radiation oncologists and medical physicists to assess whether the bladder wall dose is optimal after the radiotherapy treatment plan has been completed. To the best of our knowledge, there are no studies on how to ensure that the lowest bladder wall dose is achieved when optimizing radiotherapy treatment plans for pelvic cancer patients and how to adequately evaluate the bladder wall dose outcome of the treatment plans.

To address these challenges, this study developed a bladder wall dose prediction model based on the multiscale spatial relationship between the planning target volume (PTV) and the bladder wall or whole bladder. The model is based on the fact that the dose to a given organ at risk (OAR) is correlated with its distance relative to the PTV.^14^, ^15^, ^16^ The model provides a quantitative expectation of the radiation dose to the bladder wall before the plan is designed, allowing radiation oncologists to optimize the PTV contour, avoid unnecessary expansion of the PTV, and evaluate the quality of the radiotherapy treatment plan after completion. The model also allows medical physicists to optimize the parameters of the radiotherapy treatment plans, accurately assess the quality of the plans, minimize the bladder wall dose, and mitigate radiation damage to the patient.

## 2 MATERIALS AND METHODS

### 2.1 Patient datasets

This study was conducted in patients with rectal or gynecologic cancer, which represent the majority of pelvic tumor patients at our radiotherapy center. We studied 49 patients with rectal cancer and 45 patients with gynecological cancer whose CT images were acquired on a Siemens SOMATOM Spirit helical CT scanner with a tube voltage of 130 kV and a slice thickness of 5 mm.

### 2.2 Treatment planning system (TPS)

Treatment planning was performed in Monaco 5.40 TPS (Elekta AB, Stockholm, Sweden). The patients’ target volumes and OARs were outlined and reviewed by two associate chief radiation oncologists. Radiotherapy treatment plans were independently designed and reviewed by two medical physicist, one of whom was a senior physicist. We applied dynamic intensity‐modulated radiotherapy in the Monaco dynamic multi‐leaf collimator (dMLC) mode using the Monte Carlo algorithm. Optimization was constrained by normal tissue priority. The calculation grid spacing and statistical uncertainty were 0.3 cm and 0.7%, respectively, for each calculation. The dose volume histogram (DVH) had a resolution of 0.1 cm.

### 2.3 Bladder wall contouring

To obtain the bladder wall contour, the theoretical bladder wall thickness was first determined using the following equation, derived from the study of Fananapazir et al., which correlates the logarithm of the bladder wall thickness with the logarithm of the bladder volume.^17^

$$\mathrm{Bladder}\;\mathrm{wall}\;\mathrm{thickness}\;\;\left(\mathrm{mm}\right)=e^{\left(3.6105-0.52\times \ln V_{bladder}\left(ml\right)\right)}$$

The inner bladder wall contour was then obtained by shrinking the entire bladder contour (outer wall contour) in the three‐dimensional direction according to the calculated bladder wall thickness using the Monaco auto‐margin function. Finally, the bladder wall contour was obtained by subtracting the inner bladder wall contour from the entire bladder contour. The final bladder wall contour was reviewed and fine‐tuned by an associate chief radiation oncologist based on the actual CT images, and to minimize the influence of subjective factors, this work was divided between two physicians.

### 2.4 Multiscale spatial relationship analysis

The multiscale spatial relationship between the PTV and the bladder wall was characterized by calculating the proportion of the bladder wall volume overlapped by the $PTV_{+x\;cm}$ (structure obtained after expanding the PTV contour by x cm, x = 0, 0.5, 1, 1.5, 2, 2.5, 3). The volume of the bladder wall intersecting the $PTV_{+x\;cm}$ was obtained using the auto‐margin function of Monaco TPS. The percentage of the bladder wall volume overlapped by the $PTV_{+x\;cm}$ was calculated using the following equation:

$$\begin{array}{ccc} & & P_{bw\;in\;PTV_{+x\;cm}\;}\left(\%\right)\\ & & =V_{bw\;in\;PTV_{+x\;cm}\;}\;\left(cm^{3}\right)÷V_{bw}\left(cm^{3}\right)\times 100, \end{array}$$

where $V_{bw}\left(cm^{3}\right)$ is the volume of the bladder wall (cm^3^), $V_{bw\;in\;PTV_{+x\;cm}}\left(cm^{3}\right)$ is the volume of the bladder wall intersecting the $PTV_{+x\;cm}$, and $P_{bw\;in\;PTV_{+x\;cm}\;}\left(\%\right)$ is the percentage of the bladder wall volume overlapped by the $PTV_{+x\;cm}$.

The percentage of the bladder volume overlapped by the $PTV_{+x\;cm}$ ($P_{b\;in\;PTV_{+x\;cm}\;}\left(\%\right))$ was obtained by the same method.

### 2.5 Dose parameter selection

The absolute volume (cm^3^) of the bladder wall receiving 10−35 Gy in 5 Gy bins ($V_{10-35Gy}\;\left(cm^{3}\right)$) and the mean dose to the bladder wall (*D*_mean_(Gy)) were determined as predictors of acute urinary toxicity based on the study by Willigenburg et al.^12^ Therefore, we used the absolute $V_{xGy}\;\left(cm^{3}\right)$ (x = 10, 15, 20, 25, 30, 35, 40, 45) as the subject of model prediction in this study. To mitigate the impact of prescription dose variation, each treatment plan was systematically recalibrated to ensure that 95% of the PTV was effectively covered by the 50 Gy. Table 1 shows the distribution of all bladder wall dose parameters.

**TABLE 1:** The distribution of bladder wall dose parameters (mean, SD).

Dose parameter	Total group	Rectal cancer	Gynecologic cancer
Number of patients	94	49	45
$V_{10Gy}\left(cm^{3}\right)$	49.8 (9.3)	46.1 (7.5)	54.0 (9.5)
$V_{15Gy}\left(cm^{3}\right)$	45.9 (9.9)	41.5 (8.4)	50.7 (9.1)
$V_{20Gy}\left(cm^{3}\right)$	42.1 (10.2)	37.4 (9.0)	47.3 (8.9)
$V_{25Gy}\left(cm^{3}\right)$	38.6 (10.2)	33.7 (9.1)	43.8 (8.7)
$V_{30Gy}\left(cm^{3}\right)$	35.4 (10.1)	30.6 (9.1)	40.5 (8.6)
$V_{35Gy}\left(cm^{3}\right)$	32.4 (9.8)	28.0 (9.0)	37.2 (8.3)
$V_{40Gy}\left(cm^{3}\right)$	29.3 (9.3)	25.3 (8.8)	33.7 (7.8)
$V_{45Gy}\left(cm^{3}\right)$	25.8 (8.6)	22.4 (8.4)	29.6 (7.2)
$D_{mean}\left(\mathrm{Gy}\right)$	37.56 (5.44)	35.45 (5.99)	39.85 (3.62)

$V_{xGy}\left(cm^{3}\right)$ means the absolute volume (cm^3^) of the bladder wall receiving x Gy. $D_{mean}\left(Gy\right)$ means the mean dose to the bladder wall.

SD, standard deviation.

### 2.6 Statistical analysis

Mean and standard deviation (SD) were used to describe normally distributed data, while median and interquartile range (IQR) were used for skewed data. The paired *t*‐test was used to compare the significance of differences between the two groups of normally distributed data. The significance level was set as a *p*‐value of less than 0.05. All statistical analyses were performed using IBM SPSS Statistics (IBM Corp. Released 2019. IBM SPSS Statistics for Windows, version 26.0. Armonk, New York, USA: IBM Corp.).

We used linear regression methods to construct prediction models for bladder wall dosimetry. The parameters used for the analysis included $P_{b\;in\;PTV_{+x\;cm}}\;\left(\%\right)$, $P_{bw\;in\;PTV_{+x\;cm}}\;\left(\%\right)$, bladder volume ($V_{\mathrm{bladder}}\left(cm^{3}\right)$), bladder wall volume ($V_{bw}\left(cm^{3}\right)$), and PTV volume on the CT slices encompassing the bladder ($V_{PTV}\left(cm^{3}\right)$. All or some of these parameters were selected as independent variables for linear regression analysis, and the best combination of independent variables was selected to construct a linear regression model for bladder wall dose based on the results of the linear regression analysis. Table 2 shows the distribution of all the independent variables. For example, to construct a predictive model for $V_{30Gy}\left(cm^{3}\right)$ of the bladder wall, we performed linear regression using SPSS software with $V_{30Gy}\left(cm^{3}\right)$ as the dependent variable and all of the parameters in Table 2 as alternative independent variables. We used SPSS's three variable filtering methods: stepwise, backward, and forward (Figure S3). These methods automatically filtered the independent variables and obtained the optimal combinations for each method. To reduce the influence of the pre‐selected combinations of independent variables on the optimization results of SPSS, we repeated this process with two subsets of parameters: {$V_{PTV}\left(cm^{3}\right)$, $V_{bw}\left(cm^{3}\right)$, $V_{bladder}\left(cm^{3}\right)$, $P_{bw\;in\;PTV_{+x\;cm}}\left(\%\right)$ (x = 0, 0.5, 1, 1.5, 2, 2.5, 3)} and {$V_{PTV}\left(cm^{3}\right)$, $V_{bw}\left(cm^{3}\right)$, $V_{bladder}\left(cm^{3}\right)$, $P_{b\;in\;PTV_{+x\;cm}}\left(\%\right)$ (x = 0, 0.5, 1, 1.5, 2., 2. 5, 3)}. This resulted in a total of nine prediction models (including duplicates), which then underwent further manual screening. We verified that these models met the F‐test criterion, that each independent variable had a significant effect on the dependent variable, that there was no multicollinearity problem among the independent variables, and that the model residuals were normally distributed and homoscedastic. The coefficient of determination (*R*‐squared value, *R* ^2^) is an important metric used to evaluate linear regression models, and its value can be interpreted as *R* ^2^× 100 percent of the variation in the dependent variable is accounted for by the variation in the predictors. However, *R* ^2^ tends to increase with the number of predictors, which does not necessarily imply a better model fit. Therefore, we used adjusted *R* ^2^ to account for the effect of the number of predictors on *R* ^2^. Adjusted *R* ^2^ penalizes the addition of predictors that do not improve model fit and is inversely related to the number of predictors. We used adjusted *R* ^2^ as a criterion for selecting the best model among the nine candidates. More details on the methodology used to select the best model can be found in the Supplementary Material. The formulas for *R* ^2^ and adjusted *R* ^2^ are shown below.^18^

$$\;R^{2}=1-\frac{\sum_{i\;=\;1}^{n}{\left(y_{i}-{\hat{y}}_{i}\right)}^{2}}{\sum_{i\;=\;1}^{n}{\left(y_{i}-\bar{y}\right)}^{2}}$$

$$\mathrm{Adjusted}\;\;R^{2}=1-\left(\frac{n-1}{n-p}\right)\left(1-R^{2}\right)$$

$y_{i}$ is the observed value for the i‐th sample, ${\hat{y}}_{i}$ is the corresponding predicted value, $\bar{y}$ is the mean value for all samples, *n* is the sample size, and p−1 is the number of predictors.

**TABLE 2:** The distribution of independent variables.

Independent variables	Total group	Rectal cancer	Gynecologic cancer
$V_{PTV}\left(cm^{3}\right)$, (median, IQR)	598.5 (488.7, 762.3)	671.9 (572.4, 806.0)	549.1 (402.3, 678.8)
$V_{bw}\left(cm^{3}\right)$, (mean, SD)	52.8 (9.4)	50.1 (7.9)	55.6 (10.0)
$V_{bladder}\left(cm^{3}\right)$, (median, IQR)	324.9 (220.1, 492.0)	289.9 (193.5, 434.9)	387.2 (283.8, 526.0)
$P_{bw\;in\;PTV_{+0\;cm}}\left(\%\right)$, (mean, SD)	39.9 (13.3)	37.6 (14.0)	42.5 (12.1)
$P_{bw\;in\;PTV_{+0.5\;cm}}\left(\%\right)$, (mean, SD)	48.2 (12.9)	45.4 (13.7)	51.4 (11.3)
$P_{bw\;in\;PTV_{+1\;cm}}\left(\%\right)$, (mean, SD)	56.9 (12.6)	53.9 (13.7)	60.3 (10.4)
$P_{bw\;in\;PTV_{+1.5\;cm}}\left(\%\right)$, (mean, SD)	64.6 (12.6)	61.4 (14.1)	68.0 (9.7)
$P_{bw\;in\;PTV_{+2\;cm}}\left(\%\right)$, (mean, SD)	71.6 (12.4)	68.7 (14.3)	74.8 (9.0)
$P_{bw\;in\;PTV_{+2.5\;cm}}\left(\%\right)$, (median, IQR)	77.8 (72.2, 88.0)	75.8 (65.4, 88.3)	79.4 (74.8, 87.1)
$P_{bw\;in\;PTV_{+3\;cm}}\left(\%\right)$, (median, IQR)	83.6 (78.4, 92.9)	82.3 (72.1, 93.7)	85.4 (80.6, 91.2)
$P_{b\;in\;PTV_{+0\;cm}}\left(\%\right)$, (median, IQR)	22.1 (12.9, 38.3)	24.2 (15.2, 38.7)	20.1 (12.2, 38.2)
$P_{b\;in\;PTV_{+0.5\;cm}}\left(\%\right)$, (median, IQR)	33.1 (23.5, 48.2)	35.0 (25.8, 49.1)	32.3 (21.9, 47.6)
$P_{b\;in\;PTV_{+1\;cm}}\left(\%\right)$, (mean, SD)	48.9 (16.3)	50.1 (16.2)	47.5 (16.5)
$P_{b\;in\;PTV_{+1.5\;cm}}\left(\%\right)$, (mean, SD)	59.9 (16.0)	61.2 (16.2)	58.4 (15.9)
$P_{b\;in\;PTV_{+2\;cm}}\left(\%\right)$, (mean, SD)	69.8 (15.2)	71.1 (15.8)	68.4 (14.5)
$P_{b\;in\;PTV_{+2.5\;cm}}\left(\%\right)$, (median, IQR)	78.6 (66.7, 90.2)	82.6 (67.4, 92.4)	76.2 (66.3, 88.3)
$P_{b\;in\;PTV_{+3\;cm}}\left(\%\right)$, (median, IQR)	86.3 (75.9, 96.5)	89.7 (76.3, 97.3)	84.3 (75.9, 93.7)

$V_{PTV}\left(cm^{3}\right)$/ $V_{bw}\left(cm^{3}\right)$/ $V_{bladder}\left(cm^{3}\right)$ means the volume (cm^3^) of the PTV/bladder wall/bladder. $P_{bw\;in\;PTV_{+x\;cm}}\left(\%\right)$ or $P_{b\;in\;PTV_{+x\;cm}}\left(\%\right)$ means the percentage of the bladder wall or entire bladder volume overlapped by the $PTV_{+x\;cm}$ (structure obtained after expanding the PTV contour by x cm).

IQR, interquartile range; SD, standard deviation.

We performed linear regression analyses on the data of all patients and on the data of patients with each disease type separately. The prediction accuracy of the new patients was compared to determine which method produced a more accurate model. Root mean square error (RMSE) and mean percentage error (MPE) were used as criteria for predictive accuracy. The predictive accuracy of the model increased as the RMSE and MPE decreased.

$$\mathrm{RMSE}\;\left(P,A\right)=\sqrt{\frac{1}{n}\sum_{i=1}^{n}{\left(P_{i}-A_{i}\right)}^{2}}$$

$$\mathrm{MPE}\left(P,A\right)\;=\frac{100}{n}\;\sum_{i\;=\;1}^{n}\frac{\left|A_{i}-P_{i}\right|}{A_{i}}$$

*P* and *A* represent the predicted and actual values, separately. $P_{i}$ and $A_{i}$ refer to the predicted and actual values of the i‐th patient, respectively.

### 2.7 Prediction model validation, radiotherapy treatment plan evaluation, and optimization

We applied the developed model to predict the outcome of radiotherapy treatment plans for 12 new patients. These patients had rectal cancer (*n* = 5) or gynecologic cancer (*n* = 7) and their treatment plan outcomes were unknown. We assessed the accuracy of the prediction model by comparing the predicted and actual bladder wall dose parameters. Using IBM SPSS Statistics software, we calculated the predicted values and two types of 95% confidence intervals (CIs) for each dose parameter. The first type, the 95% CI of the mean predicted value, reflects the model's uncertainty in estimating the mean value that multiple medical physicists would obtain from repeated optimizations of the same radiotherapy plan. The second type, the 95% CI of the individual predicted value, reflects the model's uncertainty in estimating the particular value that a single medical physicist would obtain from a single optimization of a specific radiotherapy plan, which has more variability than the first type. The 95% CI of the individual predicted value contains more uncertainty than the 95% CI of the mean predicted value, and we consider actual dose parameters below the upper limit of the 95% CI of the mean predicted value to be acceptable, so we use this upper limit as the criterion for re‐optimization. If the initially calculated bladder wall dose exceeds this criterion, the plan requires further optimization based on the model prediction.

The CI of the mean predicted value can be obtained by:

$${\hat{y}}_{h}\pm t_{\left(1-\alpha /2,n-p\right)}\times \sqrt{\frac{\sum_{i\;=\;1}^{n}{\left(y_{i}-{\hat{y}}_{i}\right)}^{2}}{n-p}\times \left(X_{h}^{T}{\left(X^{T}X\right)}^{-1}X_{h}\right)}$$

The CI of the individual predicted value can be obtained by:

$$\begin{array}{ccc} & & {\hat{y}}_{h}\pm t_{\left(1-\alpha /2,n-p\right)}\\ & & \times \sqrt{\frac{\sum_{i\;=\;1}^{n}{\left(y_{i}-{\hat{y}}_{i}\right)}^{2}}{n-p}+\frac{\sum_{i\;=\;1}^{n}{\left(y_{i}-{\hat{y}}_{i}\right)}^{2}}{n-p}\times \left(X_{h}^{T}{\left(X^{T}X\right)}^{-1}X_{h}\right)} \end{array}$$

where $y_{i}$ and ${\hat{y}}_{i}$ are the observed and predicted values for the i‐th sample, respectively, i = 1, 2, …, *n*, *n* is the sample size, p−1 is the number of predictors, $X_{h}={\left(1,X_{h,1},X_{h,2},\text{…},X_{h,p-1}\right)}^{T}\;$ is the vector of predictor values for any given observation *h*, ${\hat{y}}_{h}$ is the predicted value corresponding to $X_{h}$, and $t_{\left(1-\alpha /2,n-p\right)}$ is the critical value of the Student's t‐distribution for a confidence level of 1−α.^18^

To redesign the treatment plans, the bladder wall dose parameters obtained from the prediction model were incorporated into the cost function as an optimization objective, while keeping the other optimization parameters constant. Subsequently, the results of the redesigned plans were re‐evaluated by comparing them with the predicted results to assess the feasibility of using the prediction model to evaluate and optimize radiotherapy treatment plans.

## 3 RESULTS

When linear regression analysis was performed on different patient datasets, including all patients, rectal cancer patients only, and gynecologic cancer patients only, the results were different. The results of this analysis are presented in Tables S1‐S9. Based on these results, the optimization models shown in Tables 3, 4, 5 were derived.

**TABLE 3:** Linear regression model based on all patients.

Dependent variable	Independent variable		Equation	*R* ^2^/Adjusted *R* ^2^
$V_{10Gy}\left(cm^{3}\right)$	$P_{bw\;in\;PTV_{+3\;cm}}\left(\%\right)$	$V_{bw}\left(cm^{3}\right)$	$V_{10Gy}\;\left(cm^{3}\right)=0.969\times V_{bw}\left(cm^{3}\right)+0.255\times P_{bw\;in\;PTV_{+3\;cm}}\left(\%\right)-22.688$	0.940/0.938
$V_{15Gy}\left(cm^{3}\right)$	$P_{bw\;in\;PTV_{+3\;cm}}\left(\%\right)$		$V_{15Gy}\;\left(cm^{3}\right)=0.938\times V_{bw}\left(cm^{3}\right)+0.454\times P_{bw\;in\;PTV_{+3\;cm}}\left(\%\right)-41.584$	0.901/0.899
$V_{20Gy}\left(cm^{3}\right)$	$P_{bw\;in\;PTV_{+2\;cm}}\left(\%\right)$		$V_{20Gy}\;\left(cm^{3}\right)=0.844\times V_{bw}\left(cm^{3}\right)+0.487\times P_{bw\;in\;PTV_{+2\;cm}}\left(\%\right)-37.234$	0.885/0.883
$V_{25Gy}\left(cm^{3}\right)$	$P_{bw\;in\;PTV_{+1.5\;cm}}\left(\%\right)$		$V_{25Gy}\;\left(cm^{3}\right)=0.789\times V_{bw}\left(cm^{3}\right)+0.520\times P_{bw\;in\;PTV_{+1.5\;cm}}\left(\%\right)-36.586$	0.899/0.896
$V_{30Gy}\left(cm^{3}\right)$	$P_{bw\;in\;PTV_{+1.5\;cm}}\left(\%\right)$ $P_{b\;in\;PTV_{+2\;cm}}\left(\%\right)$		$V_{30Gy}\;\left(cm^{3}\right)=0.710\times V_{bw}\left(cm^{3}\right)+0.630\times P_{bw\;in\;PTV_{+1.5\;cm}}\left(\%\right)-0.084\times P_{b\;in\;PTV_{+2\;cm}}\;\left(\%\right)-36.859$	0.921/0.918
$V_{35Gy}\left(cm^{3}\right)$	$P_{bw\;in\;PTV_{+1\;cm}}\left(\%\right)$ $P_{b\;in\;PTV_{+0.5\;cm}}\left(\%\right)$		$V_{35Gy}\;\left(cm^{3}\right)=0.653\times V_{bw}\left(cm^{3}\right)+0.628\times P_{bw\;in\;PTV_{+1\;cm}}\left(\%\right)-0.064\times P_{b\;in\;PTV_{+0.5\;cm}}\;\left(\%\right)-35.393$	0.937/0.934
$V_{40Gy}\left(cm^{3}\right)$	$P_{bw\;in\;PTV_{+1\;cm}}\left(\%\right)$ $P_{bw\;in\;PTV_{+3\;cm}}\left(\%\right)$		$V_{40Gy}\;\left(cm^{3}\right)=0.604\times V_{bw}\left(cm^{3}\right)+0.623\times P_{bw\;in\;PTV_{+1\;cm}}\left(\%\right)-0.084\times P_{bw\;in\;PTV_{+3\;cm}}\;\left(\%\right)-30.966$	0.951/0.949
$V_{45Gy}\left(cm^{3}\right)$	$P_{bw\;in\;PTV_{+0.5\;cm}}\left(\%\right)$ $P_{b\;in\;PTV_{+0\;cm}}\left(\%\right)$		$V_{45Gy}\;\left(cm^{3}\right)=0.527\times V_{bw}\left(cm^{3}\right)+0.570\times P_{bw\;in\;PTV_{+0.5\;cm}}\left(\%\right)-0.041\times P_{b\;in\;PTV_{+0\;cm}}\;\left(\%\right)-28.429$	0.960/0.959
$D_{mean}\left(\mathrm{Gy}\right)$	$P_{bw\;in\;PTV_{+1\;cm}}\left(\%\right)$ $P_{bw\;in\;PTV_{+3\;cm}}\left(\%\right)$		$D_{mean}\left(\mathrm{Gy}\right)=0.337\times P_{bw\;in\;PTV_{+1\;cm}}\left(\%\right)+0.082\times P_{bw\;in\;PTV_{+3\;cm}}\;\left(\%\right)+11.502$	0.870/0.867

$V_{xGy}\left(cm^{3}\right)$ means the absolute volume (cm^3^) of the bladder wall receiving x Gy. $D_{mean}\left(Gy\right)$ means the mean dose to the bladder wall. $V_{bw}\left(cm^{3}\right)$ means the volume (cm^3^) of the bladder wall. $P_{bw\;in\;PTV_{+x\;cm}}\left(\%\right)$ or $P_{b\;in\;PTV_{+x\;cm}}\left(\%\right)$ means the percentage of the bladder wall or entire bladder volume overlapped by the $PTV_{+x\;cm}$ (structure obtained after expanding the PTV contour by x cm).

**TABLE 4:** Linear regression model based on the rectal cancer patients only.

Dependent variable	Independent variable		Equation	*R* ^2^/Adjusted *R* ^2^
$V_{10Gy}\left(cm^{3}\right)$	$P_{b\;in\;PTV_{+3\;cm}}\left(\%\right)$	$V_{bw}\left(cm^{3}\right)$	$V_{10Gy}\;\left(cm^{3}\right)=0.916\times V_{bw}\left(cm^{3}\right)+0.316\times P_{b\;in\;PTV_{+3\;cm}}\left(\%\right)-27.127$	0.926/0.923
$V_{15Gy}\left(cm^{3}\right)$	$P_{b\;in\;PTV_{+3\;cm}}\left(\%\right)$		$V_{15Gy}\;\left(cm^{3}\right)=0.870\times V_{bw}\left(cm^{3}\right)+0.505\times P_{b\;in\;PTV_{+3\;cm}}\left(\%\right)-45.715$	0.875/0.870
$V_{20Gy}\left(cm^{3}\right)$	$P_{bw\;in\;PTV_{+1\;cm}}\left(\%\right)$ $P_{bw\;in\;PTV_{+3\;cm}}\left(\%\right)$		$V_{20Gy}\;\left(cm^{3}\right)=0.697\times V_{bw}\left(cm^{3}\right)+0.245\times P_{bw\;in\;PTV_{+1\;cm}}\left(\%\right)+0.286\times P_{bw\;in\;PTV_{+3\;cm}}\left(\%\right)-34.129$	0.881/0.873
$V_{25Gy}\left(cm^{3}\right)$	$P_{bw\;in\;PTV_{+1.5\;cm}}\left(\%\right)$		$V_{25Gy}\;\left(cm^{3}\right)=0.640\times V_{bw}\left(cm^{3}\right)+0.517\times P_{bw\;in\;PTV_{+1.5\;cm}}\left(\%\right)-30.137$	0.881/0.875
$V_{30Gy}\left(cm^{3}\right)$	$P_{bw\;in\;PTV_{+1\;cm}}\left(\%\right)$		$V_{30Gy}\;\left(cm^{3}\right)=0.551\times V_{bw}\left(cm^{3}\right)+0.551\times P_{bw\;in\;PTV_{+1\;cm}}\left(\%\right)-26.664$	0.909/0.905
$V_{35Gy}\left(cm^{3}\right)$	$P_{bw\;in\;PTV_{+1\;cm}}\left(\%\right)$		$V_{35Gy}\;\left(cm^{3}\right)=0.538\times V_{bw}\left(cm^{3}\right)+0.555\times P_{bw\;in\;PTV_{+1\;cm}}\left(\%\right)-28.891$	0.929/0.926
$V_{40Gy}\left(cm^{3}\right)$	$P_{bw\;in\;PTV_{+1\;cm}}\left(\%\right)$ $P_{bw\;in\;PTV_{+3\;cm}}\left(\%\right)$		$V_{40Gy}\;\left(cm^{3}\right)=0.496\times V_{bw}\left(cm^{3}\right)+0.644\times P_{bw\;in\;PTV_{+1\;cm}}\left(\%\right)-0.113\times P_{bw\;in\;PTV_{+3\;cm}}\;\left(\%\right)-24.936$	0.945//0.942
$V_{45Gy}\left(cm^{3}\right)$	$P_{bw\;inPTV_{+0.5\;cm}}\left(\%\right)$		$V_{45Gy}\;\left(cm^{3}\right)=0.480\times V_{bw}\left(cm^{3}\right)+0.518\times P_{bw\;in\;PTV_{+0.5\;cm}}\left(\%\right)-25.167$	0.953/0.950
$D_{mean}\left(\mathrm{Gy}\right)$	$P_{bw\;in\;PTV_{+1\;cm}}\left(\%\right)$ $P_{b\;in\;PTV_{+3\;cm}}\left(\%\right)$		$D_{mean}\left(Gy\right)=0.321\times P_{bw\;in\;PTV_{+1\;cm}}\left(\%\right)+0.117\times P_{b\;in\;PTV_{+3\;cm}}\;\left(\%\right)+8.035$	0.898/0.893

$V_{xGy}\left(cm^{3}\right)$ means the absolute volume (cm^3^) of the bladder wall receiving x Gy. $D_{mean}\left(Gy\right)$ means the mean dose to the bladder wall. $V_{bw}\left(cm^{3}\right)$ means the volume (cm^3^) of the bladder wall. $P_{bw\;in\;PTV_{+x\;cm}}\left(\%\right)$ or $P_{b\;in\;PTV_{+x\;cm}}\left(\%\right)$ means the percentage of the bladder wall or entire bladder volume overlapped by the $PTV_{+x\;cm}$ (structure obtained after expanding the PTV contour by x cm).

**TABLE 5:** Linear regression model based on the gynecologic cancer patients only.

Dependent variable	Independent variable		Equation	*R* ^2^/Adjusted *R* ^2^
$V_{10Gy}\left(cm^{3}\right)$	$P_{bw\;in\;PTV_{+3\;cm}}\left(\%\right)$	$V_{bw}\left(cm^{3}\right)$	$V_{10Gy}\;\left(cm^{3}\right)=0.960\times V_{bw}\left(cm^{3}\right)+0.118\times P_{bw\;in\;PTV_{+3\;cm}}\left(\%\right)-9.526$	0.982/0.981
$V_{15Gy}\left(cm^{3}\right)$	$P_{bw\;in\;PTV_{+3\;cm}}\left(\%\right)$		$V_{15Gy}\;\left(cm^{3}\right)=0.931\times V_{bw}\left(cm^{3}\right)+0.328\times P_{bw\;in\;PTV_{+3\;cm}}\left(\%\right)-29.276$	0.931/0.927
$V_{20Gy}\left(cm^{3}\right)$	$P_{bw\;in\;PTV_{+3\;cm}}\left(\%\right)$		$V_{20Gy}\;\left(cm^{3}\right)=0.890\times V_{bw}\left(cm^{3}\right)+0.456\times P_{bw\;in\;PTV_{+3\;cm}}\left(\%\right)-41.431$	0.905/0.900
$V_{25Gy}\left(cm^{3}\right)$	$P_{bw\;in\;PTV_{+1.5\;cm}}\left(\%\right)$		$V_{25Gy}\;\left(cm^{3}\right)=0.803\times V_{bw}\left(cm^{3}\right)+0.424\times P_{bw\;in\;PTV_{+1.5\;cm}}\left(\%\right)-29.684$	0.918/0.914
$V_{30Gy}\left(cm^{3}\right)$	$P_{bw\;in\;PTV_{+1.5\;cm}}\left(\%\right)$		$V_{30Gy}\;\left(cm^{3}\right)=0.764\times V_{bw}\left(cm^{3}\right)+0.500\times P_{bw\;in\;PTV_{+1.5\;cm}}\left(\%\right)-36.014$	0.936/0.932
$V_{35Gy}\left(cm^{3}\right)$	$P_{bw\;in\;PTV_{+1\;cm}}\left(\%\right)$		$V_{35Gy}\;\left(cm^{3}\right)=0.519\times V_{bw}\left(cm^{3}\right)+0.706\times P_{bw\;in\;PTV_{+1\;cm}}\left(\%\right)-33.376$	0.946/0.944
$V_{40Gy}\left(cm^{3}\right)$	$P_{bw\;in\;PTV_{+1\;cm}}\left(\%\right)$		$V_{40Gy}\;\left(cm^{3}\right)=0.634\times V_{bw}\left(cm^{3}\right)+0.538\times P_{bw\;in\;PTV_{+1\;cm}}\left(\%\right)-33.988$	0.956/0.954
$V_{45Gy}\left(cm^{3}\right)$	$P_{bw\;in\;PTV_{+0.5\;cm}}\left(\%\right)$		$V_{45Gy}\;\left(cm^{3}\right)=0.546\times V_{bw}\left(cm^{3}\right)+0.531\times P_{bw\;in\;PTV_{+0.5\;cm}}\left(\%\right)-28.016$	0.962/0.960
$D_{mean}\left(Gy\right)$	$P_{bw\;in\;PTV_{+1\;cm}}\left(\%\right)$		$D_{mean}\;\left(Gy\right)=0.327\times P_{bw\;in\;PTV_{+1\;cm}}\left(\%\right)+20.116$	0.887/0.884

$V_{xGy}\left(cm^{3}\right)$ means the absolute volume (cm^3^) of the bladder wall receiving x Gy. $D_{mean}\left(Gy\right)$ means the mean dose to the bladder wall. $V_{bw}\left(cm^{3}\right)$ means the volume (cm^3^) of the bladder wall. $P_{bw\;in\;PTV_{+x\;cm}}\left(\%\right)$ or $P_{b\;in\;PTV_{+x\;cm}}\left(\%\right)$ means the percentage of the bladder wall or entire bladder volume overlapped by the $PTV_{+x\;cm}$ (structure obtained after expanding the PTV contour by x cm).

No significant effect was observed for $V_{\mathrm{bladder}\;}\left(cm^{3}\right)$ and $V_{PTV\;}\left(cm^{3}\right)$ in the models shown in Tables 3, 4, 5. $V_{xGy\;}\left(cm^{3}\right)$ was significantly dependent on bladder wall volume, but $D_{mean}$ was not. Factors such as the multiscale spatial relationship of the bladder wall to the PTV ($P_{bw\;in\;PTV_{+x\;cm}}\left(\%\right)$), the multiscale spatial relationship of the bladder to the PTV ($P_{b\;in\;PTV_{+x\;cm}}\left(\%\right)$), or a combination of both significantly affected all predicted values in the study. Based on the results in Tables S1‐S9, all models passed the *F*‐test, demonstrating their significance. The multicollinearity test revealed that all variance inflation factor (VIF) values in the model were less than 5, indicating no multicollinearity problem. The model residuals were normally distributed and homoscedastic.

The models in Table 3, 4, 5 had *R* ^2^ values of 0.918±0.031, 0.911±0.029, and 0.936±0.030, respectively, indicating that the variation in the predictors explained approximately 91.8%, 91.1%, and 93.6% of the variation in the bladder wall dose for each patient dataset.^18^

**FIGURE 1:**
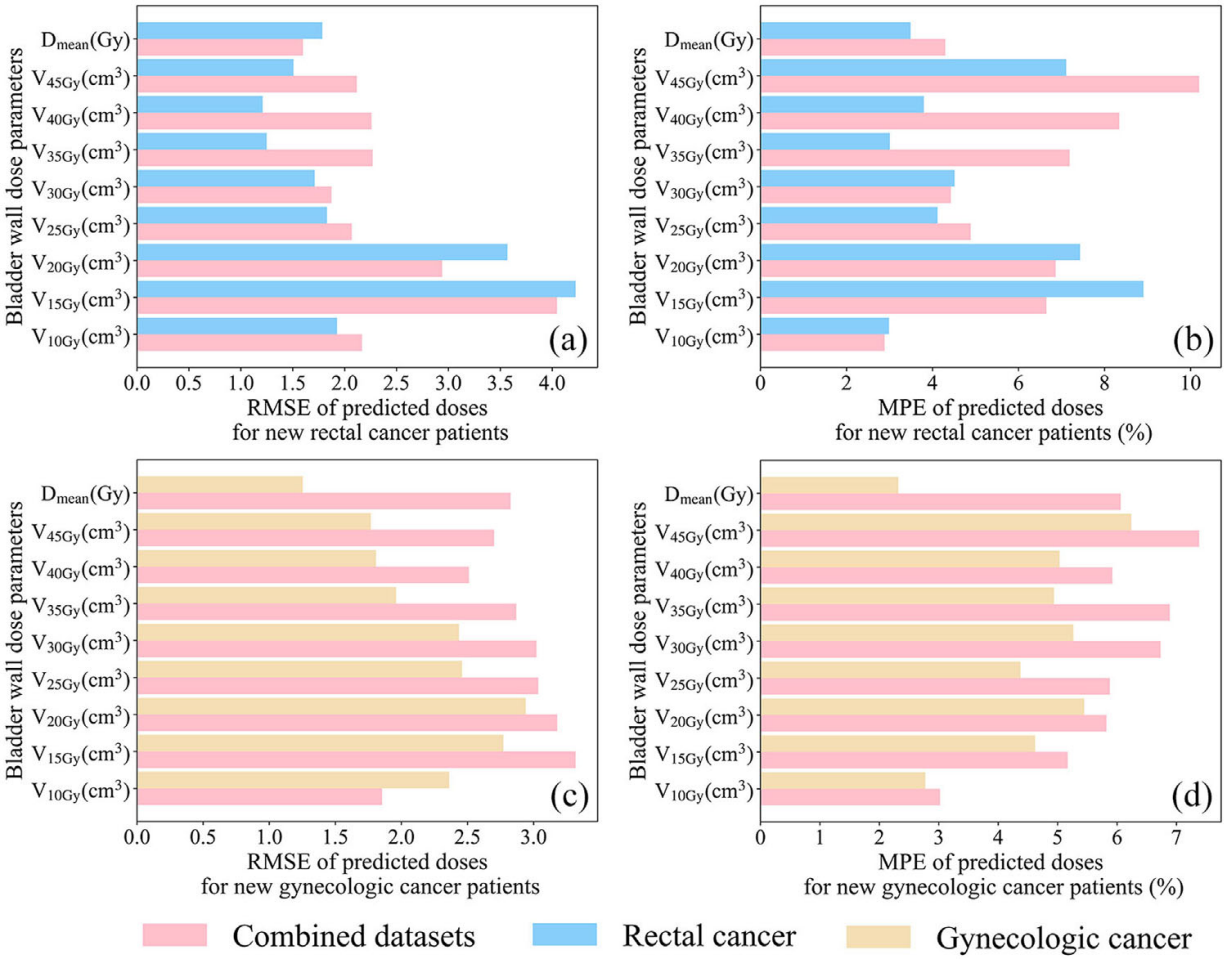
(a) RMSE and (b) MPE of predicted bladder wall doses for new rectal cancer patients based on models derived from rectal cancer patients only versus models derived from combined datasets. (c) RMSE and (d) MPE of predicted bladder wall doses for new gynecologic cancer patients based on models derived from gynecologic cancer patients only versus models derived from combined datasets. Models derived from individual disease data outperformed those derived from combined datasets.

Figure 1 presents a comparative analysis of the prediction accuracy of the model for bladder wall dose in new patients based on rectal cancer patient data and gynecologic cancer patient data, with the model derived from combined datasets serving as the benchmark. The model using rectal cancer patient data exhibited higher prediction accuracy for new rectal cancer patients, except for the RMSE values for $D_{\mathrm{mean}}\left(Gy\right)$ and $V_{xGy}\left(cm^{3}\right)$ (x = 15, 20), and the MPE values for $V_{xGy}\left(cm^{3}\right)$ (x = 10, 15, 20, 30) (Figure 1a,b). Similarly, the model using gynecologic cancer patient data showed superior prediction accuracy for new gynecologic cancer patients, except for the RMSE for $V_{10Gy\;}\left(cm^{3}\right)$ (Figure 1c,d). Therefore, in this study, linear regression models based on disease‐specific data (Tables 4 and 5) were applied to predict the bladder wall dose in new patients with corresponding diseases to evaluate their treatment plans and guide the optimization process.

**FIGURE 2:**
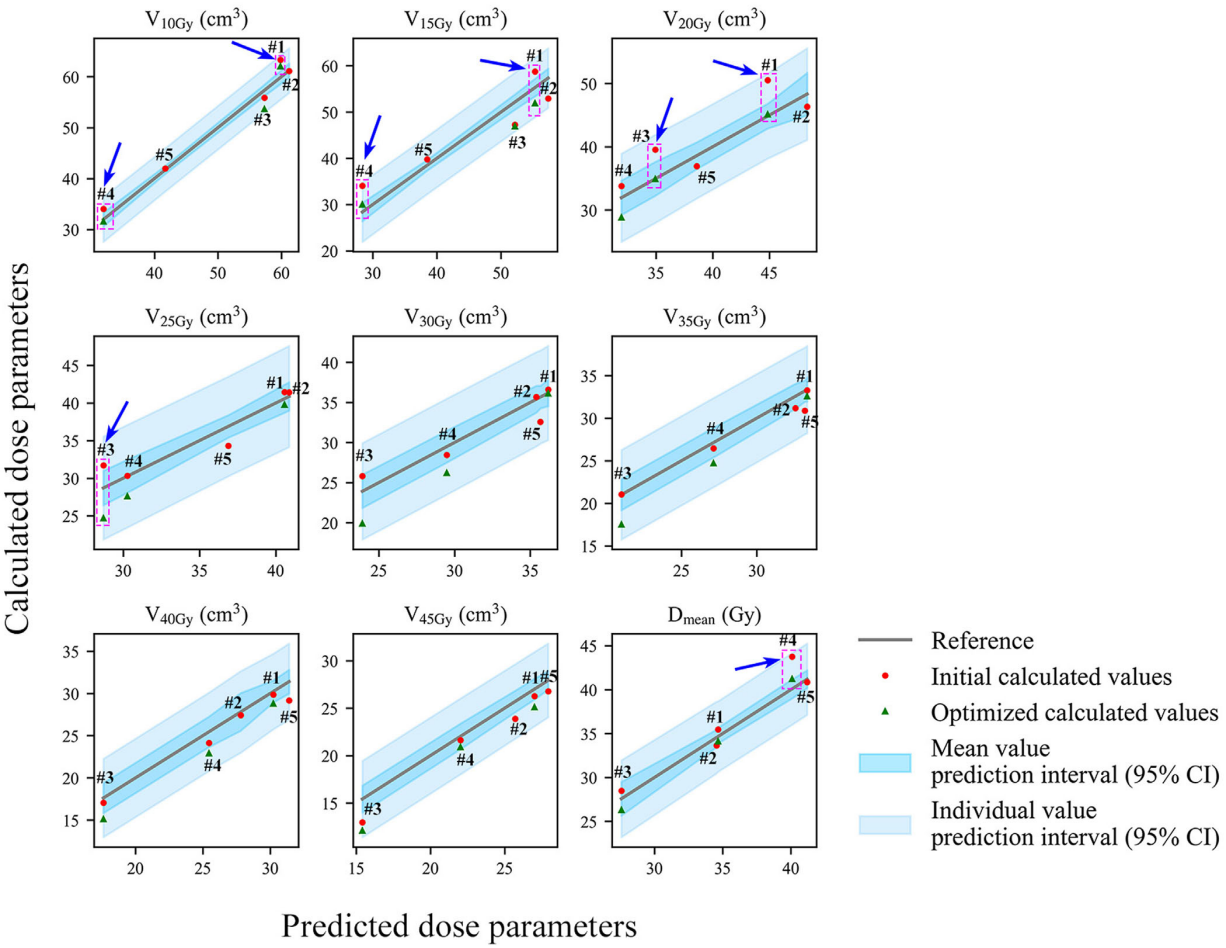
Plot of relationship between calculated and predicted values of each bladder wall dose parameter for new rectal cancer patients, both initial and after optimization. Only a few of the initially calculated values were above the upper limit of the mean prediction interval but still below the upper limit of the individual prediction interval at the 95% confidence level, and these values decreased to near or below the predicted values after the plan was redesigned. The prediction results of the model are accurate and can be used to evaluate the merits of the original radiotherapy treatment plans, help optimize the plans, and reduce the bladder wall dose to the patients.

**FIGURE 3:**
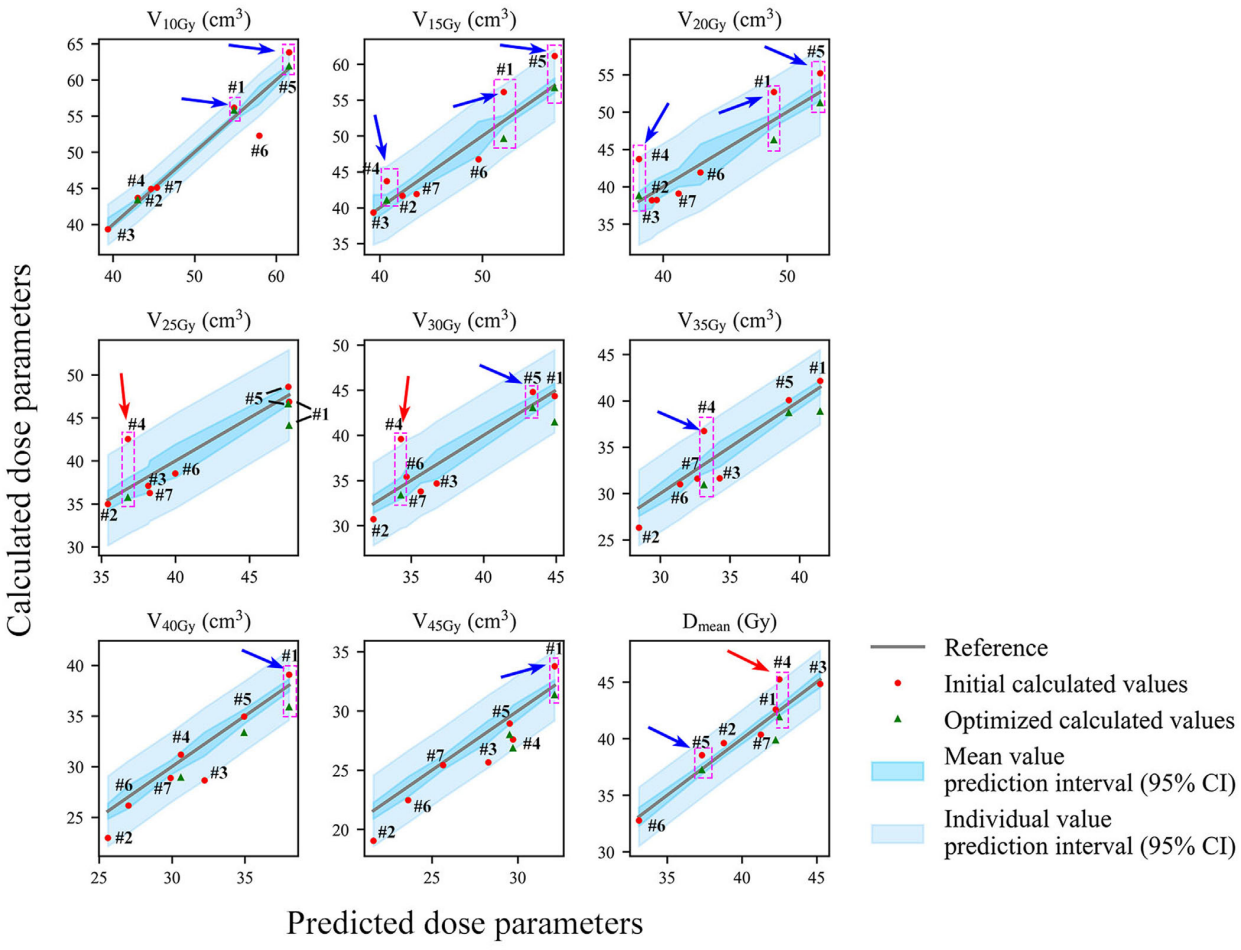
Plot of relationship between calculated and predicted values of each bladder wall dose parameter for new gynecologic cancer patients, both initial and after optimization. Only a few of the initially calculated values were above the upper limit of the mean prediction interval but still below the upper limit of the individual prediction interval at the 95% confidence level, and these values decreased to near or below the predicted values after the plan was redesigned. The prediction results of the model are accurate and can be used to evaluate the merits of the original radiotherapy treatment plans, help optimize the plans, and reduce the bladder wall dose to the patients.

For these 12 new patients, the RMSE and MPE corresponding to the predicted and observed values of $V_{10-45Gy\;}\left(cm^{3}\right)$ are evaluated to be 2.37 cm^3^ and 5.00%, respectively. For $D_{mean}\left(Gy\right)$, the analogous RMSE and MPE values are 1.50 Gy and 2.81%, respectively. Figures 2 and 3 show the actual calculated values of the bladder wall dose parameters for the new patients compared to the model predicted values. (Note: The model validation using the patient data from the training set is presented in Figures S1 and S2, which show a good overall agreement between the predicted and observed values). The gray lines represent the ideal scenario where the actual values exactly match the predicted values, while the dark blue intervals represent the model prediction intervals with 95% confidence for the mean value and the light blue intervals represent the model prediction intervals with 95% confidence for the individual value. Red dots symbolize the initial calculated values for each bladder wall dose parameter. All elevated values in Figures 2 and 3 are indicated by the blue arrows.

Figure 2 reveals that the initial calculated values of all bladder wall dose parameters for two rectal cancer patients (patients #2 and #5) were below the upper limit of the 95% CI of the corresponding mean predicted values. Therefore, no plan optimization was required for these patients. However, the following patients had some dose parameters that exceeded the upper limit of the 95% CI of the mean predicted values and required plan optimization: patient #1 ($V_{10Gy\;}\left(cm^{3}\right)$, $V_{15Gy\;}\left(cm^{3}\right)$, and $V_{20Gy\;}\left(cm^{3}\right)$), patient #3 ($V_{20Gy\;}\left(cm^{3}\right)$ and $V_{25Gy\;}\left(cm^{3}\right)$), and patient #4 ($V_{10Gy\;}\left(cm^{3}\right)$, $V_{15Gy\;}\left(cm^{3}\right)$, and $D_{mean}\left(Gy\right)$). Figure 3 demonstrates that among the seven new gynecologic cancer patients, the plans for patients #2, #3, #6, and #7 were optimal and did not require optimization. For patient #1, $V_{xGy\;}\left(cm^{3}\right)$ (x = 10, 15, 20, 40, 45) were elevated. For patient #4, $V_{xGy\;}\left(cm^{3}\right)$ (x = 15, 20, 25, 30, 35) and $D_{mean}\left(\mathrm{Gy}\right)$ were elevated. For patient #5, $V_{xGy\;}\left(cm^{3}\right)$ (x = 10, 15, 20, 30) and $D_{mean}\left(\mathrm{Gy}\right)$ were elevated. The elevated values mentioned above were all lower than the upper limit of the 95% CI for the individual predicted value, except for the values of $V_{25Gy\;}\left(cm^{3}\right)$, $V_{30Gy\;}\left(cm^{3}\right),$ and $D_{mean}\left(\mathrm{Gy}\right)$ for patient #4 (red arrows).

The cost function for the entire bladder was defined in the initial radiotherapy plan design according to our previous practice. For the plans that needed further optimization, we added the cost function for the bladder wall. The discrepancy between the actual bladder wall dose and the model predicted value was used to adjust the parameters of the bladder wall cost function. The optimized results are shown as green triangles in Figures 2 and 3. After the optimization process, all previously elevated parameters, including the $V_{25Gy\;}\left(cm^{3}\right)$, $V_{30Gy\;}\left(cm^{3}\right)$, and $D_{mean}\left(Gy\right)$ for patient #4 (gynecologic cancer), the individual with the highest deviating values, were reduced to acceptable levels (below the upper limit of the 95% CI of the mean predicted values) as delineated by the reference frame of the pink rectangular box. The $D_{mean}\left(Gy\right)$ and $V_{10-45Gy\;}\left(cm^{3}\right)$ of the bladder wall decreased by 2.27±0.80 Gy (5.8%±1.8%) and 2.96±2.05 cm^3^ (7.9%±5.4%), respectively, after optimization. The correlation coefficients between the predicted and actual dose metrics improved from 0.943±0.039 to 0.982±0.011. The differences between the optimized and initial calculated doses were statistically significant (paired *t*‐test, *p* < 0.001). In addition, Table 6 shows that the reoptimized plans met the dose‐volume criteria for the other critical OARs,^19^ with no significant deviations from the original plans (paired *t*‐test, *p* > 0.05).

**TABLE 6:** Comparison of metrics and statistical results for other critical OARs between initial and reoptimized plans.

		Bowel Space	Femoral Head‐Neck			Rectum
		$V_{45Gy}<190cm^{3}$ (initial/ reoptimized)	$V_{44Gy}<5\%$ (initial/ reoptimized)	$V_{40Gy}<35\%$ (initial/ reoptimized)	$V_{30Gy}<50\%$ (initial/ reoptimized)	$V_{50Gy}<50\%$ (initial/ reoptimized)
Rectal cancer	#1	67.1/53.7	0.3/0.5	2.7/2.4	17.0/20.0	–
	#3	12.6/11.4	0/0.1	0.2/0.5	3.9/11.3	–
	#4	121.3/118.1	1.6/1.4	5.7/5.1	24.4/30.0	–
Gynecologic cancer	#1	59.0/61.0	2.5/2.6	6.2/7.1	20.1/21.9	4.6/6
	#4	187.2/164.7	1/1.2	4.1/5.2	14.6/18.4	13.1/12
	#5	159.5/165.8	1.3/1.5	6.9/6.1	33.6/28.7	0.1/0
*p* value (Initial values vs. reoptimized values, paired *t*‐test)	p = 0.275	p = 0.175	p = 0.770	p = 0.170	p = 0.935	

$V_{xGy}<ycm^{3}$: The absolute volume of an OAR receiving a dose of x Gy or greater should be less than y cm^3^. $V_{xGy}<y\%$: The relative volume of an OAR receiving a dose of x Gy or greater should be less than y% of the total OAR volume.

## 4 DISCUSSION

We have developed a mathematical model that uses linear regression to predict bladder wall dose parameters ($V_{xGy\;}\left(cm^{3}\right)$, $D_{mean}\left(Gy\right)$) in this study. The datasets for this endeavor were derived from rectal and gynecologic cancer patients. Interestingly, models using data exclusively from either rectal or gynecologic cancer patients yielded more accurate predictions than those utilizing data from both patient groups (Figure 1). This finding could be attributed to the different design strategies implemented in the radiotherapy treatment plans for the two patient groups. For instance, rectal and bladder dose reductions are required simultaneously in gynecologic cancer patients, whereas only bladder dose reductions are required in rectal cancer patients.

In the optimized model, $V_{xGy\;}\left(cm^{3}\right)$ as the dependent variable represents the absolute volume (cm^3^) of the bladder wall receiving x Gy, and it is obvious that the bladder wall volume will be its significant influencing factor, which has a significant positive effect on it (Tables S1‐S8). In addition, for rectal cancer patients, $V_{xGy\;}\left(cm^{3}\right)$ was significantly affected only by the spatial relationship of the bladder to the PTV ($P_{b\;in\;PTV_{+1\;cm}}\left(\%\right)$) ($V_{10-15Gy\;}\left(cm^{3}\right)$) or the multiscale spatial relationship of the bladder wall to the PTV ($P_{bw\;in\;PTV_{+x\;cm}}\left(\%\right)$, x = 0.5, 1, 1.5, 3) ($V_{20-45Gy\;}\left(cm^{3}\right)$), whereas in patients with gynecologic cancers, $V_{xGy\;}\left(cm^{3}\right)$ was significantly influenced only by the $P_{bw\;in\;PTV_{+x\;cm}}\left(\%\right)$, (x = 0.5, 1, 1.5, 3) (Tables S1‐S8). The mean dose ($D_{mean}\left(Gy\right)$) was significantly affected only by the spatial relationship of the bladder or bladder wall to the PTV in both rectal and gynecologic cancer patients. In rectal cancer patients, $D_{mean}\left(Gy\right)$ was positively influenced by $P_{bw\;in\;PTV_{+1\;cm}}\left(\%\right)$ and $P_{b\;in\;PTV_{+3\;cm}}\left(\%\right)$, whereas in gynecologic cancer patients, $D_{mean}\left(Gy\right)$ was positively influenced only by $P_{bw\;in\;PTV_{+1\;cm}}\left(\%\right)$. Apparently, different bladder wall dose parameters were significantly influenced mainly by the multiscale spatial relationship between the PTV and the bladder or bladder wall, characterized by the proportion of bladder or bladder wall volume overlapped by the different distance expansions of the PTV.

The optimized models showed high *R* ^2^ values (0.911±0.029, 0.936±0.030) for both rectal and gynecologic cancer patients, indicating that the models were highly explanatory of the variation in the dependent variables.^18^ The multiscale spatial relationship between the bladder wall (or bladder) and PTV ($P_{bw\;in\;PTV_{+x\;cm}}\left(\%\right)$ or $P_{b\;in\;PTV_{+x\;cm}}\left(\%\right)$) could account for most of the variation in bladder wall dose. Therefore, to minimize radiation damage to the bladder wall, it is incumbent upon the radiation oncologist to limit unnecessary contour expansion of the PTV near the bladder side when delineating the patient's target volume.

The predictive accuracy of the model was demonstrated by the proximity of the initial calculated values for new patients to the predicted values of the model. In addition, radiotherapy treatment plans with initial calculated values exceeding the upper limit of the mean prediction interval (95% CI) were redesigned according to the model's prediction results. The optimized results fell below the upper limit of the mean prediction interval (95% CI) and showed a significant reduction in bladder wall dose (*p* < 0.001). This suggests that our proposed model is capable of predicting bladder wall dose prior to planning, guiding the setting of planning parameters, and providing individualized evaluation criteria for bladder wall dose in pelvic tumor patients. Furthermore, it can assess whether the bladder wall dose is excessive after planning, thereby guiding the redesign of radiotherapy treatment plans for patients with high bladder wall doses.

The accuracy of our prediction model is competitive with the results of other related studies. Moore et al.^20^ developed a mean dose prediction model based on the geometric relationship between OAR and PTV in head and neck and prostate cancer patients. The study showed a reduction in bladder dose after optimization, a result that is mirrored in our study. Yang et al.^14^ constructed a linear regression model for prostate cancer patients that used the L15 of the bladder (the distance of expansion of the PTV corresponding to 15% of the bladder volume overlapping with the expanded PTV) to predict the D15 (dose at 15% volume on the bladder dose‐volume histogram). After optimization, the mean dose to the bladder was reduced by 1.57±1.52 Gy, compared to a reduction of 2.27±0.80 Gy in our study. Ma et al.^21^ used a deep convolutional neural network to predict dose distribution in prostate cancer patients with a mean absolute error of 0.029±0.020 (mean PTV doses were normalized to 1), slightly higher than our result of approximately 0.016±0.012. Based on the 3D dose prediction models acquired from deep learning, the percentage errors of the mean values of $V_{30Gy}\left(\%\right)$ and $V_{40Gy}\left(\%\right)$ of the bladder wall predicted by Adabi et al. were 2.69% and 2.76%, respectively,^22^ and the results of Koike et al. (for the whole bladder) were 6.51% and 4.82%, respectively,^23^ while ours were 0.24% and 3.96%, respectively.

In conclusion, the developed model provides a robust basis for guiding rational delineation of PTV, setting optimization parameters of radiotherapy treatment plans, guiding optimization and evaluation of the treatment plans, and predicting bladder toxicity in patients with pelvic tumors. As in the study by Moore et al.,^15^ the multiscale spatial relationship based on OAR and PTV for predicting bladder wall dose could potentially be extended to all major OARs involved in different radiotherapy treatment plans. This would provide objective optimization parameters for automated radiotherapy treatment planning. Compared to current deep neural network‐based methods for predicting patient DVH,^21^, ^22^, ^23^, ^24^, ^25^ this method could potentially maintain accuracy while having lower hardware requirements and being easier to implement and scale.

Nevertheless, our study has several limitations. First, the relatively small sample size should be improved by accumulating more data or by multicenter collaboration, which would increase the accuracy and robustness of the prediction model. Second, the manual processing of patient data was time‐consuming, a problem that could be alleviated in the future by using scripting programs to automate the process, thereby improving efficiency. Finally, the generalizability of the model needs to be further validated, as it was developed exclusively with data from our center. In the next step, we will develop a program based on Python to realize the construction of prediction models and the presentation of prediction results automatically and quickly. Using Python libraries such as pydicom, numpy, OpenCV, etc., we can achieve scaling of contours, acquisition of volumes and various dose parameters by automatically analyzing and processing the CT images, RT structures, and DVH results exported from the TPS. And the construction of linear regression prediction models can also be realized using statsmodels or sklearn in Python. The constructed program will be used in multicenter studies for training and validation with more data.

## 5 CONCLUSION

We proposed a novel method to predict and estimate bladder wall dose in pelvic tumor patients based on the multiscale spatial relationship between OAR and PTV and multiple linear regression analysis. The model demonstrated high predictive accuracy. It can help radiation oncologists to estimate bladder wall dose after target volume delineation, medical physicists to optimize and evaluate radiotherapy treatment plans, and patients to reduce bladder toxicity and improve quality of life by minimizing bladder wall dose.

## AUTHOR CONTRIBUTIONS

Xiang Gao conceived the experiments. Xiang Gao acquired and analyzed the data for the work. Lei Ge and Junfeng Gao contoured and checked the outlines of OARs. Xiang Gao and Zheng Cao designed and optimized the treatment plans. Xiang Gao and Zheng Cao designed the study and analyzed the result. Lei Ge, Junfeng Gao, and Zheng Cao participated in writing manuscript. The final version of the manuscript has been reviewed and approved for publication by all authors.

## CONFLICT OF INTEREST STATEMENT

The authors declare no conflicts of interest.

## Supporting information

Figure S1 Scatter plot illustrating the agreement between the observed and predicted values of each bladder wall dose parameter for rectal cancer patients in the training set. The actual values are close to the predicted values for the training data set.

Figure S2 Scatter plot illustrating the agreement between the observed and predicted values of each bladder wall dose parameter for gynecologic cancer patients in the training set. The actual values are close to the predicted values for the training data set.

Figure S3 Setup screen for linear regression analysis in SPSS software.

Supporting Information

Supporting Information

## Data Availability

The original contributions presented in the study are included in the article/Supplementary Material. Further inquiries can be directed to the corresponding authors.
