# Supplementary figures and images for: Multiscale spatial relationship‐based model for predicting bladder wall dose in pelvic radiotherapy
Source: J Appl Clin Med Phys. 2023 Sep 12;25(2):e14153. doi: 10.1002/acm2.14153 (PMC10860549; doi:10.1002/acm2.14153)

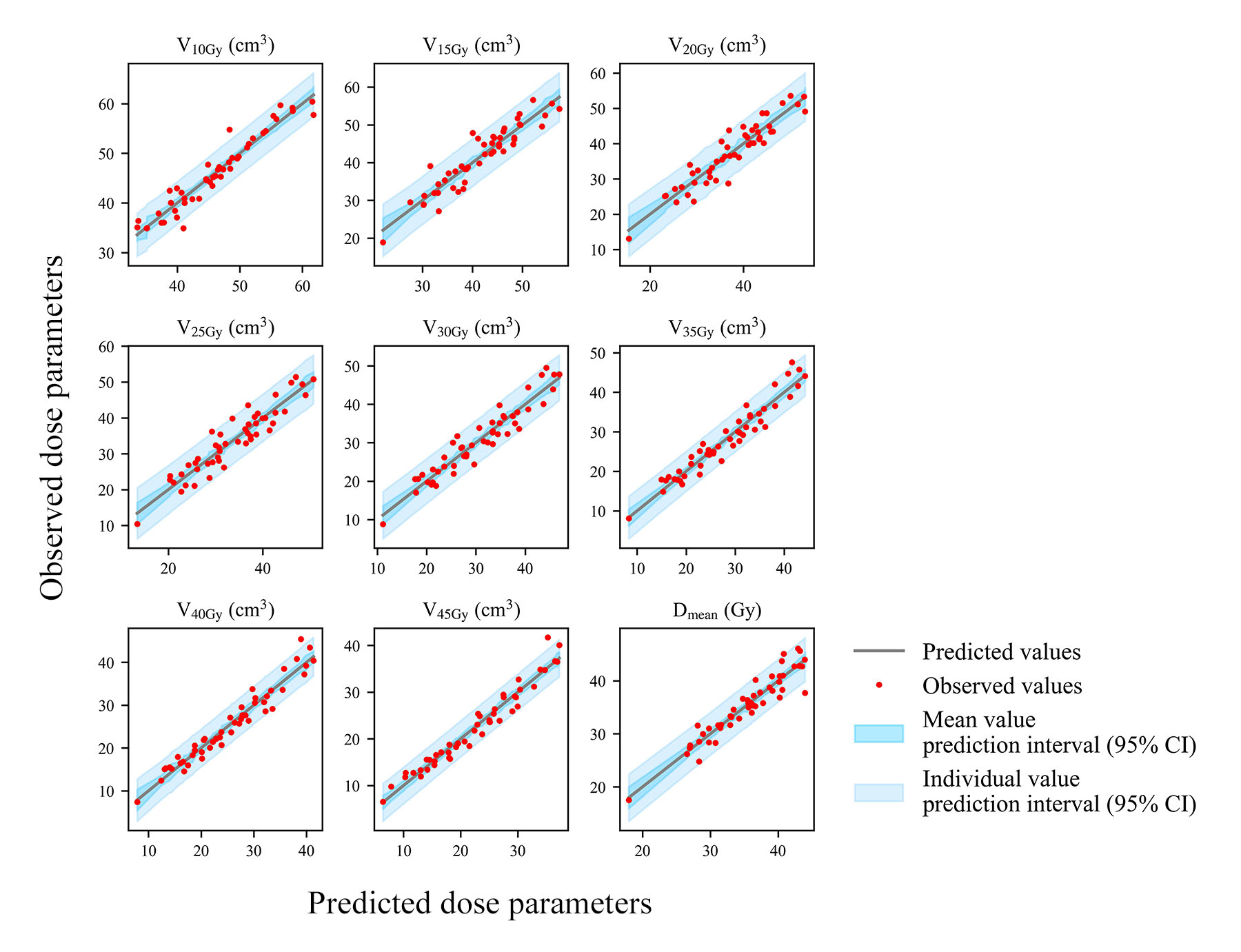

Supplement: Supplementary file 1 — Figure S1 Scatter plot illustrating the agreement between the observed and predicted values of each bladder wall dose parameter for rectal cancer patients in the training set. The actual values are close to the predicted values for the training data set. [file ACM2-25-e14153-s002.jpg]

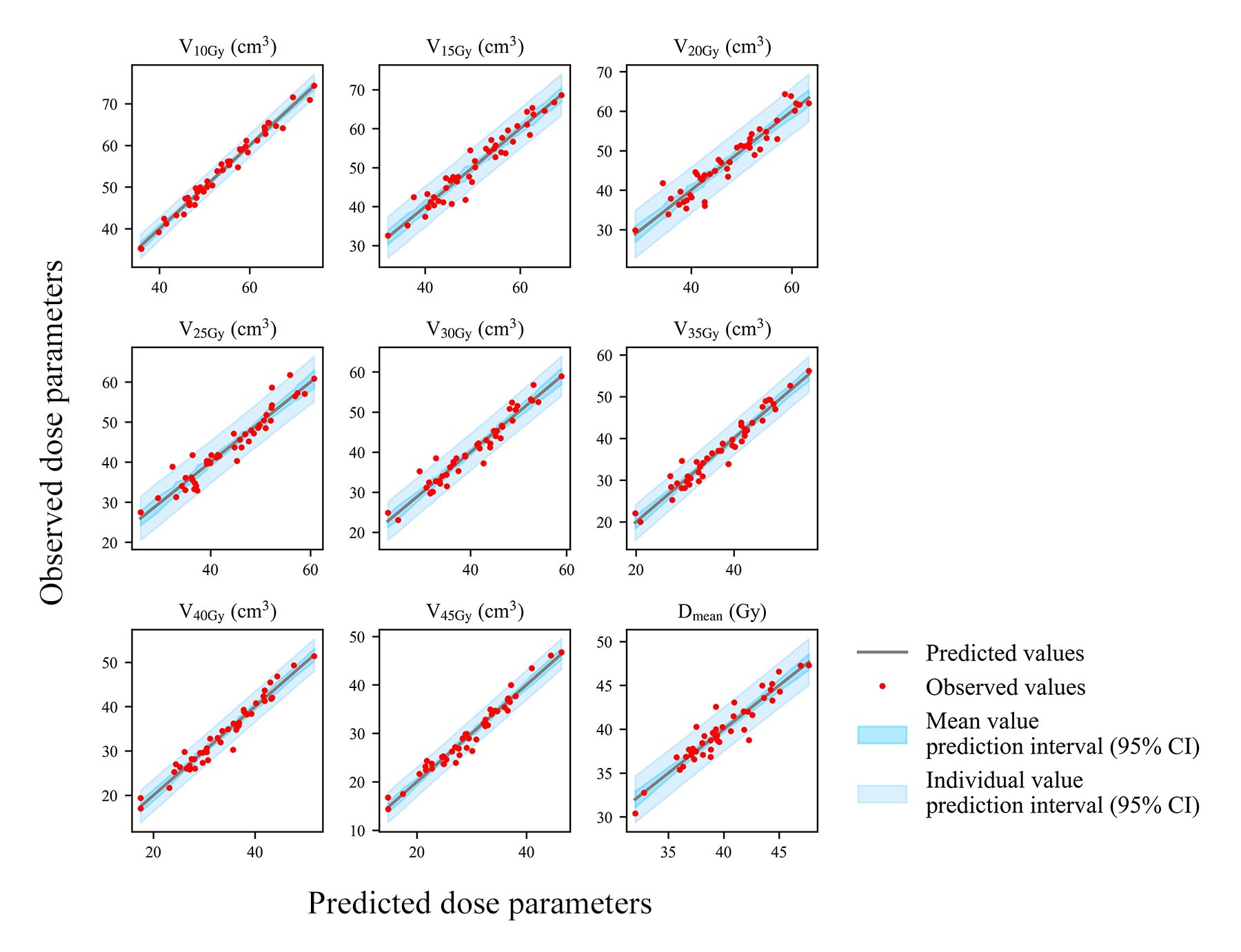

Supplement: Supplementary file 2 — Figure S2 Scatter plot illustrating the agreement between the observed and predicted values of each bladder wall dose parameter for gynecologic cancer patients in the training set. The actual values are close to the predicted values for the training data set. [file ACM2-25-e14153-s005.jpg]

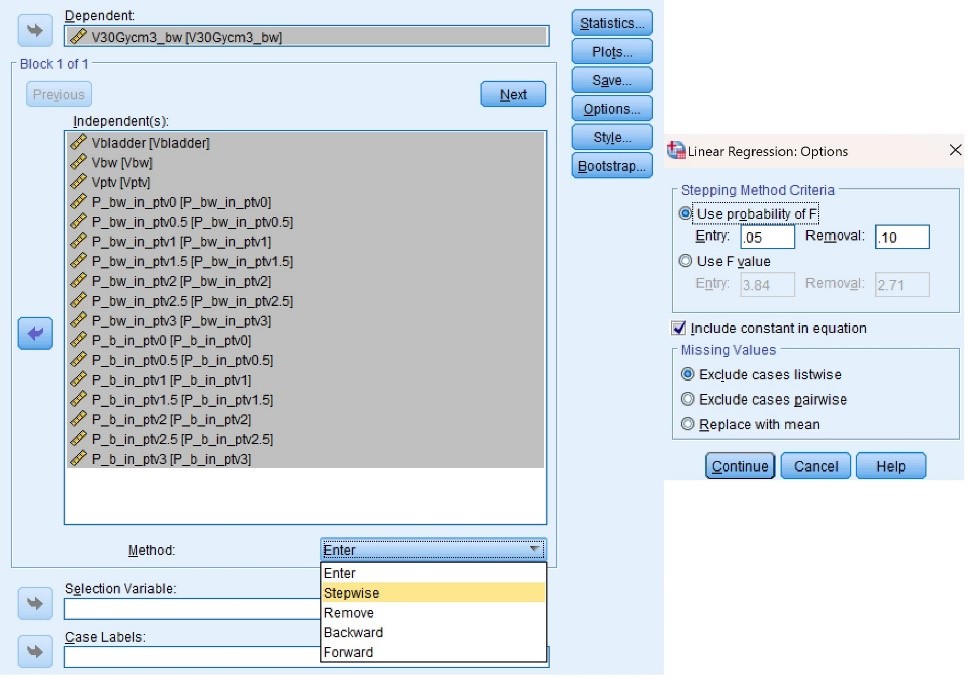

Supplement: Supplementary file 3 — Figure S3 Setup screen for linear regression analysis in SPSS software. [file ACM2-25-e14153-s004.jpg]
