# Supplementary material for: Multiscale spatial relationship‐based model for predicting bladder wall dose in pelvic radiotherapy
Source: J Appl Clin Med Phys. 2023 Sep 12;25(2):e14153. doi: 10.1002/acm2.14153 (PMC10860549; doi:10.1002/acm2.14153)
Supplement: Supplementary file 4 — Supporting Information [file ACM2-25-e14153-s001.docx]

**The details of the methodology used to select the best prediction model (an example of the prediction of** $\boldsymbol{V}_{\boldsymbol{30}\boldsymbol{Gy}}\boldsymbol{(}\boldsymbol{cm}^{\boldsymbol{3}}\boldsymbol{)}$ **based on gynecologic cancer patient data)**

1. A brief overview of the forward, backward, and stepwise method for variable selection in linear regression analysis using SPSS software.

All three methods of independent variable selection were automated in the SPSS software.

The **forward** method works by sequentially entering the predictors into the model. The first variable considered is the one that has the highest positive or negative correlation with the outcome. This is entered if it meets the inclusion criteria. Next, the predictor with the highest partial correlation that has not yet been entered is evaluated. Entry stops when none of the remaining variables meet the threshold.

In contrast, the **backward** method first includes all variables, which are then sequentially removed. The predictor with the smallest partial correlation with the outcome is first considered for elimination. If it meets the elimination criteria, it is removed. Next, the remaining variable with the smallest partial correlation is evaluated. This continues until no variables meet the removal criteria.

The **stepwise** method combines forward and backward selection. At each step, the currently excluded predictor with the lowest F probability is entered if it is sufficiently small. Variables already in the model are removed when their F-probabilities become sufficiently large. The process ends when no more entries or deletions are possible.

Inclusion and removal are determined by the significance (probability) of the F value, with inclusion below the entry value (default 0.05) and removal above the removal value (default 0.1).


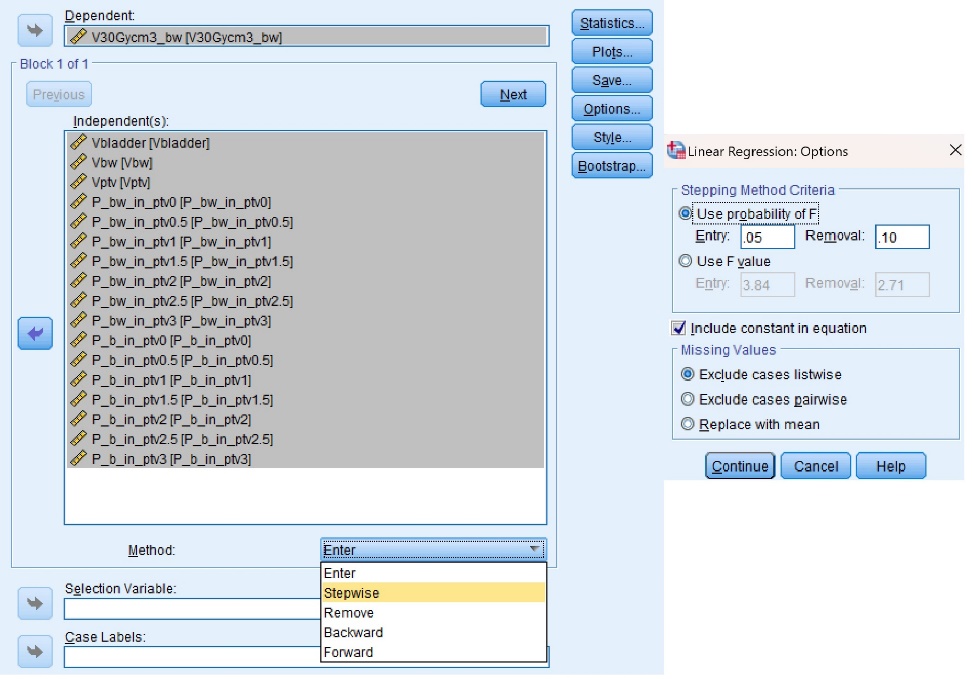


Figure S3 Setup screen for linear regression analysis in SPSS software.

2. To mitigate the effects of the initial combination of alternative predictor variables on the results of variable selection in SPSS, three different sets of alternative predictors were constructed: (1) {$V_{PTV}({cm}^{3})$, $V_{bw}({cm}^{3})$, $V_{bladder}({cm}^{3})$, $P_{bw in {PTV}_{+x cm}}(\%)$, $P_{b in {PTV}_{+x cm}}(\%)$}, (2) {$V_{PTV}({cm}^{3})$, $V_{bw}({cm}^{3})$, $V_{bladder}({cm}^{3})$, $P_{bw in {PTV}_{+x cm}}(\%)$}, and (3) {$V_{PTV}({cm}^{3})$, $V_{bw}({cm}^{3})$, $V_{bladder}({cm}^{3})$, $P_{b in {PTV}_{+x cm}}(\%)$} (x = 0, 0.5, 1, 1.5, 2., 2. 5, 3).

3. For the set of variables (1), the optimal models obtained by the forward, backward, and stepwise methods were all $V_{30Gy}\left( {cm}^{3} \right)=0.764\times V_{bw}\left( {cm}^{3} \right)+0.500\times P_{bw in {PTV}_{+1.5 cm}}\left( \% \right)-36.014$, and the values of adjusted R^2^ were all 0.932.

4. For the set of variables (2), the optimal models obtained by the forward, backward, and stepwise methods were all $V_{30Gy}\left( {cm}^{3} \right)=0.764\times V_{bw}\left( {cm}^{3} \right)+0.500\times P_{bw in {PTV}_{+1.5 cm}}\left( \% \right)-36.014$，and the values of adjusted R^2^ were all 0.932.

5. For the set of variables (3), the optimal models obtained by the forward and stepwise methods were all $V_{30Gy}\left( {cm}^{3} \right)=0.819\times V_{bw}\left( {cm}^{3} \right)+0.274\times P_{b in {PTV}_{+1.5 cm}}\left( \% \right)-21.071$，and the values of adjusted R^2^ were all 0.846. The optimal models obtained by the backward method was $V_{30Gy}\left( {cm}^{3} \right)=0.590\times V_{bw}\left( {cm}^{3} \right)+0.016\times V_{bladder}\left( {cm}^{3} \right)+0.493\times P_{b in {PTV}_{+1.5 cm}}\left( \% \right)-0.198\times P_{b in {PTV}_{+0 cm}}\left( \% \right)-22.592$，and the value of adjusted R^2^ was 0.862.

6. We verified that all these models met the F-test criterion, that each independent variable had a significant effect on the dependent variable, that there was no multicollinearity problem among the independent variables, and that the model residuals were normally distributed and homoscedastic.

7. Automatic variable screening was performed using SPSS software, which generated three different predictive models based on the three sets of variables using forward, backward, and stepwise methods, respectively. The optimal model was then selected manually by contrasting the adjusted R^2^ values. Clearly, “$V_{30Gy}\left( {cm}^{3} \right)=0.764\times V_{bw}\left( {cm}^{3} \right)+0.500\times P_{bw in {PTV}_{+1.5 cm}}\left( \% \right)-36.014$” had the largest adjusted R^2^ (0.932) and was selected as the final optimal model.
