# Supplementary material for: Multiscale spatial relationship‐based model for predicting bladder wall dose in pelvic radiotherapy
Source: J Appl Clin Med Phys. 2023 Sep 12;25(2):e14153. doi: 10.1002/acm2.14153 (PMC10860549; doi:10.1002/acm2.14153)
Supplement: Supplementary file 5 — Supporting Information [file ACM2-25-e14153-s003.docx]

**Supplementary tables and captions**

Table S1 Linear regression analysis of the dependent variable $V_{10Gy}({cm}^{3})$.

|  |  | Unstandardized Coefficients | | Standardized Coefficients | t | VIF |
| --- | --- | --- | --- | --- | --- | --- |
|  |  | B | SE | Beta |  |  |
| Total group | Constant | -22.688 | 2.444 | - | -9.282*** | - |
|  | $P_{bw in {PTV}_{+3 cm}}(\%)$ | 0.255 | 0.021 | 0.314 | 12.004*** | 1.031 |
|  | $V_{bw}\left( {cm}^{3} \right)$ | 0.969 | 0.026 | 0.974 | 37.258*** | 1.031 |
|  | R^2^/ Adjusted R^2^ | 0.940/0.938 | | | | |
|  | F | F (2,91) = 709.664, p<0.001 | | | | |
| Rectal cancer | Constant | -27.127 | 3.330 | - | -8.146*** | - |
|  | $P_{b in {PTV}_{+3 cm}}(\%)$ | 0.316 | 0.025 | 0.534 | 12.724*** | 1.095 |
|  | $V_{bw}\left( {cm}^{3} \right)$ | 0.916 | 0.039 | 0.973 | 12.724*** | 1.095 |
|  | R^2^/ Adjusted R^2^ | 0.926/0.923 | | | | |
|  | F | F (2,46) = 288.215, p<0.001 | | | | |
| Gynecologic cancer | Constant | -9.526 | 2.970 | - | -3.207** ^a^ | - |
|  | $P_{bw in {PTV}_{+3 cm}}(\%)$ | 0.118 | 0.028 | 0.094 | 4.268*** | 1.125 |
|  | $V_{bw}\left( {cm}^{3} \right)$ | 0.960 | 0.021 | 1.018 | 45.998*** | 1.125 |
|  | R^2^/ Adjusted R^2^ | 0.982/0.981 | | | | |
|  | F | F (2, 42) = 1126.971, p<0.001 | | | | |

SE: standard error. VIF: variance inflation factor. **: p<0.01. ***: p<0.001. a: p=0.003. $V_{10Gy}({cm}^{3})$ means the absolute volume (cm^3^) of the bladder wall receiving 10 Gy. $P_{bw in {PTV}_{+3 cm}}(\%)$ means the percentage of the bladder wall volume overlapped by the ${PTV}_{+3 cm}$ (structure obtained after expanding the PTV contour by 3 cm). $P_{b in {PTV}_{+3 cm}}(\%)$ means the percentage of the bladder volume overlapped by the ${PTV}_{+3 cm}$. $V_{bw}\left( {cm}^{3} \right)$ means the volume (cm^3^) of the bladder wall.

Table S2 Linear regression analysis of the dependent variable $V_{15Gy}({cm}^{3})$.

|  |  | Unstandardized Coefficients | | Standardized Coefficients | t | VIF |
| --- | --- | --- | --- | --- | --- | --- |
|  |  | B | SE | Beta |  |  |
| Total group | Constant | -41.584 | 3.328 | - | -12.496*** | - |
|  | $P_{bw in {PTV}_{+3 cm}}(\%)$ | 0.454 | 0.029 | 0.525 | 15.662*** | 1.031 |
|  | $V_{bw}({cm}^{3})$ | 0.938 | 0.035 | 0.888 | 26.469*** | 1.031 |
|  | R^2^/ Adjusted R^2^ | 0.901/0.899 | | | | |
|  | F | F (2,91) = 413.145, p<0.001 | | | | |
| Rectal cancer | Constant | -45.715 | 4.892 | - | -9.344*** | - |
|  | $P_{b in {PTV}_{+3 cm}}(\%)$ | 0.505 | 0.036 | 0.755 | 13.852*** | 1.095 |
|  | $V_{bw}({cm}^{3})$ | 0.870 | 0.058 | 0.817 | 14.986*** | 1.095 |
|  | R^2^/ Adjusted R^2^ | 0.875/0.870 | | | | |
|  | F | F (2,46) = 161.145, p<0.001 | | | | |
| Gynecologic cancer | Constant | -29.276 | 5.588 | - | -5.239*** | - |
|  | $P_{bw in {PTV}_{+3 cm}}(\%)$ | 0.328 | 0.052 | 0.273 | 6.338*** | 1.125 |
|  | $V_{bw}({cm}^{3})$ | 0.931 | 0.039 | 1.021 | 23.696*** | 1.125 |
|  | R^2^/ Adjusted R^2^ | 0.931/0.927 | | | | |
|  | F | F (2, 42) = 282.126, p<0.001 | | | | |

SE: standard error. VIF: variance inflation factor. ***: p<0.001. $V_{15Gy}({cm}^{3})$ means the absolute volume (cm^3^) of the bladder wall receiving 15 Gy. $P_{bw in {PTV}_{+3 cm}}(\%)$ means the percentage of the bladder wall volume overlapped by the ${PTV}_{+3 cm}$ (structure obtained after expanding the PTV contour by 3 cm). $P_{b in {PTV}_{+3 cm}}(\%)$ means the percentage of the bladder volume overlapped by the ${PTV}_{+3 cm}$. $V_{bw}({cm}^{3})$ means the volume (cm^3^) of the bladder wall.

Table S3 Linear regression analysis of the dependent variable $V_{20Gy}({cm}^{3})$.

|  |  | Unstandardized Coefficients | | Standardized Coefficients | t | VIF |
| --- | --- | --- | --- | --- | --- | --- |
|  |  | B | SE | Beta |  |  |
| Total group | Constant | -37.234 | 3.051 | - | -12.202*** | - |
|  | $P_{bw in {PTV}_{+2 cm}}(\%)$ | 0.487 | 0.029 | 0.594 | 16.686*** | 1.006 |
|  | $V_{bw}({cm}^{3})$ | 0.844 | 0.039 | 0.778 | 21.835*** | 1.006 |
|  | R^2^/ Adjusted R^2^ | 0.885/0.883 | | | | |
|  | F | F (2,91) = 351.197, p<0.001 | | | | |
| Rectal cancer | Constant | -34.129 | 5.221 | - | -6.536*** | - |
|  | $P_{bw in {PTV}_{+1 cm}}(\%)$ | 0.245 | 0.073 | 0.373 | 3.346** ^a^ | 4.699 |
|  | $P_{bw in {PTV}_{+3 cm}}(\%)$ | 0.286 | 0.074 | 0.444 | 3.883*** | 4.934 |
|  | $V_{bw}({cm}^{3})$ | 0.697 | 0.064 | 0.617 | 10.866*** | 1.218 |
|  | R^2^/ Adjusted R^2^ | 0.881/0.873 | | | | |
|  | F | F (2,46) = 110.905, p<0.001 | | | | |
| Gynecologic cancer | Constant | -41.431 | 6.361 | - | -6.513*** | - |
|  | $P_{bw in {PTV}_{+3 cm}}(\%)$ | 0.456 | 0.059 | 0.391 | 7.730*** | 1.125 |
|  | $V_{bw}({cm}^{3})$ | 0.890 | 0.045 | 1.007 | 19.918*** | 1.125 |
|  | R^2^/ Adjusted R^2^ | 0.905/0.900 | | | | |
|  | F | F (2, 42) = 199.032, p<0.001 | | | | |

SE: standard error. VIF: variance inflation factor. **: p<0.01. ***: p<0.001. a: p=0.002. $V_{20Gy}({cm}^{3})$ means the absolute volume (cm^3^) of the bladder wall receiving 20 Gy. $P_{bw in {PTV}_{+x cm}}(\%)$ means the percentage of the bladder wall volume overlapped by the ${PTV}_{+x cm}$ (structure obtained after expanding the PTV contour by x cm). $V_{bw}({cm}^{3})$ means the volume (cm^3^) of the bladder wall.

Table S4 Linear regression analysis of the dependent variable $V_{25Gy}({cm}^{3})$.

|  |  | Unstandardized Coefficients | | Standardized Coefficients | t | VIF |
| --- | --- | --- | --- | --- | --- | --- |
|  |  | B | SE | Beta |  |  |
| Total group | Constant | -36.586 | 2.667 | - | -13.719*** | - |
|  | $P_{bw in {PTV}_{+1.5 cm}}(\%)$ | 0.520 | 0.027 | 0.641 | 19.190*** | 1.001 |
|  | $V_{bw}({cm}^{3})$ | 0.789 | 0.036 | 0.721 | 21.609*** | 1.001 |
|  | R^2^/ Adjusted R^2^ | 0.899/0.896 | | | | |
|  | F | F (2,91) = 403.705, p<0.001 | | | | |
| Rectal cancer | Constant | -30.137 | 3.733 | - | -8.072*** | - |
|  | $P_{bw in {PTV}_{+1.5 cm}}(\%)$ | 0.517 | 0.033 | 0.803 | 15.713*** | 1.007 |
|  | $V_{bw}({cm}^{3})$ | 0.640 | 0.059 | 0.558 | 10.921*** | 1.007 |
|  | R^2^/ Adjusted R^2^ | 0.881/0.875 | | | | |
|  | F | F (2,46) = 169.741, p<0.001 | | | | |
| Gynecologic cancer | Constant | -29.684 | 3.813 | - | -7.785*** | - |
|  | $P_{bw in {PTV}_{+1.5 cm}}(\%)$ | 0.424 | 0.040 | 0.474 | 10.542*** | 1.034 |
|  | $V_{bw}({cm}^{3})$ | 0.803 | 0.039 | 0.923 | 20.542*** | 1.034 |
|  | R^2^/ Adjusted R^2^ | 0.918/0.914 | | | | |
|  | F | F (2, 42) = 234.902, p<0.001 | | | | |

SE: standard error. VIF: variance inflation factor. ***: p<0.001. $V_{25Gy}({cm}^{3})$ means the absolute volume (cm^3^) of the bladder wall receiving 25 Gy. $P_{bw in {PTV}_{+1.5 cm}}(\%)$ means the percentage of the bladder wall volume overlapped by the ${PTV}_{+1.5 cm}$ (structure obtained after expanding the PTV contour by 1.5 cm). $V_{bw}({cm}^{3})$ means the volume (cm^3^) of the bladder wall.

Table S5 Linear regression analysis of the dependent variable $V_{30Gy}({cm}^{3})$.

|  |  | Unstandardized Coefficients | | Standardized Coefficients | t | VIF |
| --- | --- | --- | --- | --- | --- | --- |
|  |  | B | SE | Beta |  |  |
| Total group | Constant | -36.859 | 2.660 | - | -13.855*** | - |
|  | $P_{bw in {PTV}_{+1.5 cm}}(\%)$ | 0.630 | 0.046 | 0.786 | 13.803*** | 3.688 |
|  | $P_{b in {PTV}_{+2 cm}}\left( \% \right)$ | -0.084 | 0.040 | -0.126 | -2.104* ^a^ | 4.101 |
|  | $V_{bw}({cm}^{3})$ | 0.710 | 0.037 | 0.658 | 19.106*** | 1.348 |
|  | R^2^/ Adjusted R^2^ | 0.921/0.918 | | | | |
|  | F | F (2,91) = 349.094, p<0.001 | | | | |
| Rectal cancer | Constant | -26.664 | 3.057 | - | -8.723*** | - |
|  | $P_{bw in {PTV}_{+1 cm}}(\%)$ | 0.551 | 0.030 | 0.828 | 18.587*** | 1.000 |
|  | $V_{bw}({cm}^{3})$ | 0.551 | 0.051 | 0.481 | 10.791*** | 1.000 |
|  | R^2^/ Adjusted R^2^ | 0.909/0.905 | | | | |
|  | F | F (2,46) = 229.128, p<0.001 | | | | |
| Gynecologic cancer | Constant | -36.014 | 3.324 | - | -10.833*** | - |
|  | $P_{bw in {PTV}_{+1.5 cm}}(\%)$ | 0.500 | 0.035 | 0.568 | 14.251*** | 1.034 |
|  | $V_{bw}({cm}^{3})$ | 0.764 | 0.034 | 0.893 | 22.418*** | 1.034 |
|  | R^2^/ Adjusted R^2^ | 0.936/0.932 | | | | |
|  | F | F (2, 42) = 304.727, p<0.001 | | | | |

SE: standard error. VIF: variance inflation factor. *: p<0.05. ***: p<0.001. a: p=0.038. $V_{30Gy}({cm}^{3})$ means the absolute volume (cm^3^) of the bladder wall receiving 30 Gy. $P_{bw in {PTV}_{+x cm}}(\%)$ means the percentage of the bladder wall volume overlapped by the ${PTV}_{+x cm}$ (structure obtained after expanding the PTV contour by x cm). $P_{b in {PTV}_{+2 cm}}(\%)$means the percentage of the bladder volume overlapped by the ${PTV}_{+2 cm}$ (structure obtained after expanding the PTV contour by 2 cm). $V_{bw}({cm}^{3})$ means the volume (cm^3^) of the bladder wall.

Table S6 Linear regression analysis of the dependent variable $V_{35Gy}({cm}^{3})$.

|  |  | Unstandardized Coefficients | | Standardized Coefficients | t | VIF |
| --- | --- | --- | --- | --- | --- | --- |
|  |  | B | SE | Beta |  |  |
| Total group | Constant | -35.393 | 1.904 | - | -18.595*** | - |
|  | $P_{bw in {PTV}_{+1 cm}}(\%)$ | 0.628 | 0.036 | 0.807 | 17.275*** | 3.095 |
|  | $P_{b in {PTV}_{+0.5 cm}}(\%)$ | -0.064 | 0.029 | -0.105 | -2.198* ^a^ | 3.250 |
|  | $V_{bw}({cm}^{3})$ | 0.653 | 0.030 | 0.625 | 21.974*** | 1.146 |
|  | R^2^/ Adjusted R^2^ | 0.937/0.934 | | | | |
|  | F | F (2,91) = 442.863, p<0.001 | | | | |
| Rectal cancer | Constant | -28.891 | 2.673 | - | -10.809*** | - |
|  | $P_{bw in {PTV}_{+1 cm}}(\%)$ | 0.555 | 0.026 | 0.843 | 21.428*** | 1.000 |
|  | $V_{bw}({cm}^{3})$ | 0.538 | 0.045 | 0.474 | 12.052*** | 1.000 |
|  | R^2^/ Adjusted R^2^ | 0.929/0.926 | | | | |
|  | F | F (2,46) = 299.852, p<0.001 | | | | |
| Gynecologic cancer | Constant | -33.376 | 2.657 | - | -12.560*** | - |
|  | $P_{bw in {PTV}_{+1 cm}}(\%)$ | 0.706 | 0.030 | 0.855 | 23.442*** | 1.037 |
|  | $V_{bw}({cm}^{3})$ | 0.519 | 0.029 | 0.653 | 17.898*** | 1.037 |
|  | R^2^/ Adjusted R^2^ | 0.946/0.944 | | | | |
|  | F | F (2, 42) = 368.892, p<0.001 | | | | |

SE: standard error. VIF: variance inflation factor. *: p<0.05. ***: p<0.001. a: p=0.030. $V_{35Gy}({cm}^{3})$ means the absolute volume (cm^3^) of the bladder wall receiving 35 Gy. $P_{bw in {PTV}_{+1 cm}}(\%)$ means the percentage of the bladder wall volume overlapped by the ${PTV}_{+1 cm}$ (structure obtained after expanding the PTV contour by 1 cm). $P_{b in {PTV}_{+0.5 cm}}(\%)$ means the percentage of the bladder volume overlapped by the ${PTV}_{+0.5 cm}$ (structure obtained after expanding the PTV contour by 0.5 cm). $V_{bw}({cm}^{3})$ means the volume (cm^3^) of the bladder wall.

Table S7 Linear regression analysis of the dependent variable $V_{40Gy}({cm}^{3})$.

|  |  | Unstandardized Coefficients | | Standardized Coefficients | t | VIF |
| --- | --- | --- | --- | --- | --- | --- |
|  |  | B | SE | Beta |  |  |
| Total group | Constant | -30.966 | 2.596 | - | -11.927*** | - |
|  | $P_{bw in {PTV}_{+1 cm}}(\%)$ | 0.623 | 0.037 | 0.842 | 16.766*** | 4.610 |
|  | $P_{bw in {PTV}_{+3 cm}}(\%)$ | -0.084 | 0.041 | -0.103 | -2.023* ^a^ | 4.755 |
|  | $V_{bw}({cm}^{3})$ | 0.604 | 0.025 | 0.607 | 24.382*** | 1.134 |
|  | R^2^/ Adjusted R^2^ | 0.951/0.949 | | | | |
|  | F | F (2,91) = 579.216, p<0.001 | | | | |
| Rectal cancer | Constant | -24.936 | 3.480 | - | -7.166*** | - |
|  | $P_{bw in {PTV}_{+1 cm}}(\%)$ | 0.644 | 0.049 | 0.999 | 13.226*** | 4.699 |
|  | $P_{bw in {PTV}_{+3 cm}}(\%)$ | -0.113 | 0.049 | -0.179 | -2.310* ^b^ | 4.934 |
|  | $V_{bw}({cm}^{3})$ | 0.496 | 0.043 | 0.446 | 11.598*** | 1.218 |
|  | R^2^/ Adjusted R^2^ | 0.945//0.942 | | | | |
|  | F | F (2,46) = 259.592, p<0.001 | | | | |
| Gynecologic cancer | Constant | -33.988 | 2.259 | - | -15.049*** | - |
|  | $P_{bw in {PTV}_{+1 cm}}(\%)$ | 0.538 | 0.025 | 0.717 | 21.808*** | 1.037 |
|  | $V_{bw}({cm}^{3})$ | 0.634 | 0.026 | 0.814 | 24.747*** | 1.037 |
|  | R^2^/ Adjusted R^2^ | 0.956/0.954 | | | | |
|  | F | F (2, 42) = 458.496, p<0.001 | | | | |

SE: standard error. VIF: variance inflation factor. *: p<0.05. ***: p<0.001. a: p=0.046. b: p=0.026. $V_{40Gy}({cm}^{3})$ means the absolute volume (cm^3^) of the bladder wall receiving 40 Gy. $P_{bw in {PTV}_{+x cm}}(\%)$ means the percentage of the bladder wall volume overlapped by the ${PTV}_{+x cm}$ (structure obtained after expanding the PTV contour by x cm). $V_{bw}({cm}^{3})$ means the volume (cm^3^) of the bladder wall.

Table S8 Linear regression analysis of the dependent variable $V_{45Gy}({cm}^{3})$.

|  |  | Unstandardized Coefficients | | Standardized Coefficients | t | VIF |
| --- | --- | --- | --- | --- | --- | --- |
|  |  | B | SE | Beta |  |  |
| Total group | Constant | -28.429 | 1.257 | - | -22.617*** | - |
|  | $P_{bw in {PTV}_{+0.5 cm}}(\%)$ | 0.570 | 0.024 | 0.852 | 23.968*** | 2.844 |
|  | $P_{b in {PTV}_{+0 cm}}(\%)$ | -0.041 | 0.020 | -0.073 | -2.022* ^a^ | 2.926 |
|  | $V_{bw}({cm}^{3})$ | 0.527 | 0.020 | 0.572 | 26.194*** | 1.073 |
|  | R^2^/ Adjusted R^2^ | 0.960/0.959 | | | | |
|  | F | F (2,91) = 720.738, p<0.001 | | | | |
| Rectal cancer | Constant | -25.167 | 1.911 | - | -13.171*** | - |
|  | $P_{bw in {PTV}_{+0.5 cm}}(\%)$ | 0.518 | 0.020 | 0.845 | 26.283*** | 1.002 |
|  | $V_{bw}({cm}^{3})$ | 0.480 | 0.034 | 0.453 | 14.082*** | 1.002 |
|  | R^2^/ Adjusted R^2^ | 0.953/0.950 | | | | |
|  | F | F (2,46) = 461.733, p<0.001 | | | | |
| Gynecologic cancer | Constant | -28.016 | 1.796 | - | -15.596*** | - |
|  | $P_{bw in {PTV}_{+0.5 cm}}(\%)$ | 0.531 | 0.020 | 0.825 | 26.658*** | 1.056 |
|  | $V_{bw}({cm}^{3})$ | 0.546 | 0.022 | 0.755 | 24.399*** | 1.056 |
|  | R^2^/ Adjusted R^2^ | 0.962/0.960 | | | | |
|  | F | F (2, 42) = 531.074, p<0.001 | | | | |

SE: standard error. VIF: variance inflation factor. *: p<0.05. ***: p<0.001. a: p=0.046. $V_{45Gy}({cm}^{3})$ means the absolute volume (cm^3^) of the bladder wall receiving 45 Gy. $P_{bw in {PTV}_{+0.5 cm}}(\%)$ means the percentage of the bladder wall volume overlapped by the ${PTV}_{+0.5 cm}$ (structure obtained after expanding the PTV contour by 0.5 cm). $P_{b in {PTV}_{+0 cm}}(\%)$ means the percentage of the bladder volume overlapped by the PTV. $V_{bw}({cm}^{3})$ means the volume (cm^3^) of the bladder wall.

Table S9 Linear regression analysis of the dependent variable $D_{mean}(Gy)$.

|  |  | Unstandardized Coefficients | | Standardized Coefficients | t | VIF |
| --- | --- | --- | --- | --- | --- | --- |
|  |  | B | SE | Beta |  |  |
| Total group | Constant | 11.502 | 1.711 | - | 6.724*** | - |
|  | $P_{bw in {PTV}_{+1 cm}}(\%)$ | 0.337 | 0.034 | 0.779 | 10.050*** | 4.194 |
|  | $P_{bw in {PTV}_{+3 cm}}(\%)$ | 0.082 | 0.037 | 0.172 | 2.223* ^a^ | 4.194 |
|  | R^2^/ Adjusted R^2^ | 0.870/0.867 | | | | |
|  | F | F (2,91) = 303.920, p<0.001 | | | | |
| Rectal cancer | Constant | 8.035 | 2.148 | - | 3.740*** | - |
|  | $P_{bw in {PTV}_{+1 cm}}(\%)$ | 0.321 | 0.037 | 0.733 | 8.615*** | 3.261 |
|  | $P_{b in {PTV}_{+3 cm}}(\%)$ | 0.117 | 0.040 | 0.246 | 2.893** ^b^ | 3.261 |
|  | R^2^/ Adjusted R^2^ | 0.898/0.893 | | | | |
|  | F | F (2,46) = 202.305, p<0.001 | | | | |
| Gynecologic cancer | Constant | 20.116 | 1.089 | - | 18.465*** | - |
|  | $P_{bw in {PTV}_{+1 cm}}(\%)$ | 0.327 | 0.018 | 0.942 | 18.375*** | 1.000 |
|  | R^2^/ Adjusted R^2^ | 0.887/0.884 | | | | |
|  | F | F (2, 42) = 337.640, p<0.001 | | | | |

SE: standard error. VIF: variance inflation factor. *: p<0.05. **: p<0.01. ***: p<0.001. a: p=0.029. b: p=0.006. $D_{mean}(Gy)$ means the mean dose to the bladder wall. $P_{bw in {PTV}_{+x cm}}(\%)$ means the percentage of the bladder wall volume overlapped by the ${PTV}_{+x cm}$ (structure obtained after expanding the PTV contour by x cm). $P_{b in {PTV}_{+3 cm}}(\%)$ means the percentage of the bladder volume overlapped by the ${PTV}_{+3 cm}$.
